# Molecular and functional characterization of an evolutionarily conserved CREB‐binding protein in the *Lymnaea* CNS

**DOI:** 10.1096/fj.202101225RR

**Published:** 2022-10-17

**Authors:** Dai Hatakeyama, Hiroshi Sunada, Yuki Totani, Takayuki Watanabe, Ildikó Felletár, Adam Fitchett, Murat Eravci, Aikaterini Anagnostopoulou, Ryosuke Miki, Ayano Okada, Naoya Abe, Takashi Kuzuhara, Ildikó Kemenes, Etsuro Ito, György Kemenes

**Affiliations:** ^1^ Sussex Neuroscience School of Life Sciences, University of Sussex Brighton UK; ^2^ Faculty of Pharmaceutical Sciences Tokushima Bunri University Tokushima Japan; ^3^ Kagawa School of Pharmaceutical Sciences, Tokushima Bunri University Sanuki Japan; ^4^ Department of Biology Waseda University Tokyo Japan; ^5^ Laboratory of Neuroethology Sokendai‐Hayama Hayama Japan; ^6^ Present address: Advanced Medicine, Innovation and Clinical Research Centre Tottori University Hospital Yonago Japan; ^7^ Present address: School of Life Sciences University of Westminster London UK

**Keywords:** cerebral giant cell, CREB‐binding protein, histone acetyltransferase, long‐term memory, *Lymnaea*, synaptic plasticity

## Abstract

In eukaryotes, CREB‐binding protein (CBP), a coactivator of CREB, functions both as a platform for recruiting other components of the transcriptional machinery and as a histone acetyltransferase (HAT) that alters chromatin structure. We previously showed that the transcriptional activity of cAMP‐responsive element binding protein (CREB) plays a crucial role in neuronal plasticity in the pond snail *Lymnaea stagnalis*. However, there is no information on the molecular structure and HAT activity of CBP in the *Lymnaea* central nervous system (CNS), hindering an investigation of its postulated role in long‐term memory (LTM). Here, we characterize the *Lymnaea* CBP (LymCBP) gene and identify a conserved domain of LymCBP as a functional HAT. Like CBPs of other species, LymCBP possesses functional domains, such as the KIX domain, which is essential for interaction with CREB and was shown to regulate LTM. In‐situ hybridization showed that the staining patterns of LymCBP mRNA in CNS are very similar to those of *Lymnaea* CREB1. A particularly strong LymCBP mRNA signal was observed in the cerebral giant cell (CGC), an identified extrinsic modulatory interneuron of the feeding circuit, the key to both appetitive and aversive LTM for taste. Biochemical experiments using the recombinant protein of the LymCBP HAT domain showed that its enzymatic activity was blocked by classical HAT inhibitors. Preincubation of the CNS with such inhibitors blocked cAMP‐induced synaptic facilitation between the CGC and an identified follower motoneuron of the feeding system. Taken together, our findings suggest a role for the HAT activity of LymCBP in synaptic plasticity in the feeding circuitry.

**Abbreviations**
ApCBP: *Aplysia* CBP
B4CL: B4 cluster
C/EBP: CCAAT/enhancer binding protein.
CBB: Coomassie Brilliant Blue
CBP: CREB‐binding protein
CGC: cerebral giant cell
CID: collision‐induced dissociation
CNS: central nervous system
CREB: cAMP‐responsive element binding protein
DMSO: dimethyl sulfoxide
DTT: dithiothreitol
EPSP: excitatory postsynaptic potential
ESI: electrospray ionization
HAT: histone acetyltransferase
hATF4: human activating transcription 4
IAA: iodoacetamide
IP: immunoprecipitation
IPTG: β‐d‐thiogalactopyranoside
KID: kinase inducible domain
LC/MS: liquid chromatography‐mass spectrometry
LTM: long‐term memory
LTP: long‐term potentiation
LymCBP: *Lymnaea* CBP
pCAF: p300/CBP‐associated factor
qPCR: quantitative PCR
RT: reverse transcription
SDS‐PAGE: sodium dodecyl‐sulfate polyacrylamide gel electrophoresis
Taz: transcriptional adapter zinc‐binding
TBST: TBS solution with Tween‐20

## 1 INTRODUCTION

De novo gene transcription is required for consolidation of long‐term memory (LTM)^1^ and cAMP‐responsive element binding protein (CREB)‐dependent gene expression is one of the crucial steps of this process in mammals^2^, ^3^ as well as invertebrates.^4^, ^5^, ^6^ Transcriptional activation requires the recruitment of multifunctional coactivators, resulting in a variety of different types of epigenetic modifications of histones and genomic DNA.^7^ In CREB‐dependent control of gene expression, CREB‐binding protein (CBP) functions as a coactivator of CREB. To date, CBP has been reported to be critical for LTM consolidation in mammals^8^, ^9^, ^10^ and the gastropod *Aplysia*.^5^, ^11^ One of the most important functions of CBP is due to its histone acetyltransferase (HAT) activity, which stimulates gene transcription.^12^ Several studies have shown that histone acetylation by CBP is necessary for hippocampal long‐term potentiation (LTP).^9^, ^10^, ^11^, ^12^, ^13^ Pharmacological inhibition of CBP HAT activity using the inhibitors curcumin and garcinol was reported to block memory consolidation^14^, ^15^, ^16^ and memory‐associated neuronal plasticity.^17^ These studies demonstrated that the HAT activity of CBP plays an essential role in regulating synaptic plasticity associated with memory consolidation.

The pond snail *Lymnaea stagnalis* is a widely used organism to understand evolutionarily conserved molecular mechanisms of the consolidation of LTM for taste‐related associations.^18^, ^19^, ^20^, ^21^, ^22^, ^23^ Sadamoto et al. first succeeded in the cloning of an isoform of CREB from the central nervous system (CNS) of *Lymnaea* and defined it as LymCREB1.^24^ They identified 7 different isoforms of LymCREB1 by alternative splicing and found that aversive taste conditioning significantly increased LymCREB1 gene expression.^25^ Ribeiro et al. showed that appetitive taste conditioning selectively increased phosphorylated LymCREB1 in the “learning ganglia” (the cerebral and buccal ganglia) of the *Lymnaea* CNS.^26^

An identified extrinsic modulatory neuron type of the feeding system, the cerebral giant cell (CGC) was reported to play crucial roles in LTM after both aversive and appetitive taste conditioning.^18^, ^27^, ^28^ Injection of LymCREB1 siRNA into the CGCs reduced the amplitude of excitatory postsynaptic potential (EPSP) in their identified monosynaptic follower neurons, the B1 motoneurons, suggesting that regulation of gene expression by LymCREB1 was required for synaptic enhancement in memory consolidation.^29^ Based on these previous findings, we hypothesized that the HAT activity of the *Lymnaea* CBP (LymCBP) plays an important role in the LymCREB1 initiated molecular processes of synaptic consolidation.

In the present study, we first cloned the LymCBP cDNA from the *Lymnaea* CNSs and identified neurons expressing the LymCBP mRNA. Focusing on the CGC, we pharmacologically and electrophysiologically analyzed the relationship between the postulated HAT activity of LymCBP and the synaptic plasticity involved in the aversively conditioned feeding behavior of *Lymnaea*. Our findings provide new insights into the functions of LymCBP in synaptic plasticity underlying the consolidation of associative LTM.

## 2 MATERIALS AND METHODS

### 2.1 TaqMan‐based quantitative PCR (qPCR)

To quantify the absolute copy number of LymCBP mRNA in several *Lymnaea* tissues, we performed TaqMan‐based qPCR by establishing RNA standard curves derived from a dilution series of known template concentrations. The procedures for this experiment were modified from previous reports.^30^, ^31^, ^32^ Partial LymCBP gene (from 303rd to 722nd nucleotides, which contains the open reading flame of the *N*‐terminal region) was amplified by RT‐PCR with primers shown in Table S1 (No. 15, 16), and the PCR product was subcloned into pGEM®‐T Easy Vector (Promega). The plasmid was purified with Plasmid Midi Kit (QIAGEN) and digested with restriction enzyme *Spe* I. The LymCBP RNA was synthesized with MAXIscript™ (ThermoFisher), purified with RNeasy® Mini Kit (QIAGEN), quantified the concentration, and used as the standard RNA. Serially diluted standard RNA (5 × 10^1^–5 × 10^6^ copies/μl) was reverse‐transcribed in 10 μl of the reaction mixture to prepare the first strand cDNA with 2.5 μM of the specific primer for LymCBP diluted to 1:5 and used as the standard cDNAs.

Total RNA from different types of *Lymnaea* tissue (CNS, buccal mass, penis, ovotestis, gut, and mantle) was purified with TRIzol™ Reagent (ThermoFisher). Reverse transcription (RT) of standard series (5 × 10^1^–5 × 10^6^ copies) and total RNA of *Lymnaea* tissues (each 300 ng) were performed in the buffer containing 0.2 μg yeast tRNA, 1× PCR Buffer II (ThermoFisher), 5.5 mM MgCl_2_, 0.5 mM dNTP, 0.2 μM gene‐specific primer for LymCBP, 0.3 unit/ml Prime RNase inhibitor (Eppendorf), and 0.9 units/ml MultiScribe™ Reverse Transcriptase (ThermoFisher) for 10 min at 25°C, 30 min at 48°C, and 5 min at 95°C. All RT samples were diluted 5 times by adding RNase‐free distilled water. The diluted RT solution of samples and standards (1 μl) were added to PCR‐reaction solution [final concentration: 1× TaqMan® Gene Expression Master Mix (ThermoFisher), 50 nM each primer, and 50 nM probe]. Nucleotide sequences of primers and probes were summarized in Table S1 (No. 17–20). The reaction was carried out at 95°C for 10 min, 45 cycles of 95°C for 15 s, and 60°C for 1 min with a real‐time PCR system (StepOnePlus Real‐Time PCR System, ThermoFisher). In the assay, several doses of standard cDNA (1 × 10^1^–1 × 10^6^ copies) were applied in triplicate to estimate inter‐assay coefficients of variation between runs.

### 2.2 In situ hybridization of LymCBP

The procedures for this experiment were modified from previous reports.^30^, ^31^, ^33^ Non‐functional region (from 278th to 1146th of nucleotide sequence) of LymCBP was isolated by RT‐PCR [see Table S1 (No. 21, 22) for nucleotide sequences of primers]. After insertion of this PCR product into pGEM®‐T Easy Vector, the plasmid was linearized with *Eco*R V and *Bam*H I to generate digoxigenin‐labeled antisense and sense RNA probes, respectively. The procedures for generating the probes, hybridization, and coloring were the same as those that were previously reported.^32^, ^33^ Our main target in these experiments was the paired cerebral giant cells that can be easily and accurately identified visually even without staining them with a dye or a specific probe. This is because they are the largest neurons on the ventral side of the anterior lobes of the cerebral ganglia and occupy a very characteristic position in it, as shown in the diagram in Figure 3F.

### 2.3 Western blotting, co‐precipitation, and mass spectrometry

#### 2.3.1 Experimental animals

Pond snails, *Lymnaea stagnalis*, were bred at the University of Sussex, Brighton, United Kingdom and were maintained in large holding tanks containing copper‐free water at 18–20°C on a 12‐h light‐dark cycle. The animals were fed vegetable‐based fish food (Tetra‐Phyll; TETRA Werke, Melle, Germany) twice a week and lettuce three times a week.

#### 2.3.2 Preparation of snail CNS samples for western blotting, co‐precipitation, and mass spectrometry

The whole CNS of four‐month‐old snails was dissected. Briefly, the shell of the snail was removed, and the preparation was then pinned down in a Sylgard‐coated dish containing HEPES buffered saline and dissected under a stereomicroscope (E‐Zoom6, Edmund Optics, Barrington NJ, USA). The CNS was accessed by a dorsal body incision and isolated from the buccal mass by severing all the peripheral nerves and immediately placed in Eppendorf tubes on dry ice and stored at −80°C.

#### 2.3.3 Western blotting

*Lymnaea* CNS lysates were prepared in RIPA buffer. Protein (10–20 μg) was electrophoresed on homemade 6% Bis‐Tris protein gels and transferred to polyvinylidene difluoride (PVDF) membrane. The polyclonal antibody to anti‐human‐CBP (C20, sc‐583, Santa Cruz) was used at 1:500 dilution in 4% skimmed milk in TBS‐Tween overnight at 4°C with rotation. Following washes with TBS‐Tween (10 min × 3), the membranes were incubated with the anti‐rabbit‐IgG conjugated with horseradish peroxidase (Sigma–Aldrich) at room temperature at 1:10 000 dilution for 2 h. Finally, the membranes were cleaned from the excess secondary antibody by washing with TBS‐Tween (10 min × 3) and TBS (10 min × 1). Detection of the blot was carried out with a LumiSensor™ HRP Substrate Kit (GenScript Technology).

#### 2.3.4 Immunoprecipitation assay and immunoblotting

Sixty pooled *Lymnae*a CNS samples were homogenized in RIPA lysis buffer supplemented with 2 mM PMSF, 1 mmol/L Na_3_VO_4_, and protease inhibitors, (sc‐24948, Santa Cruz) using a micropestelle. Fifty microliters of the pre‐immunoprecipitated lysate was saved as “input” to be used for immunoblotting. The homogenate was pre‐cleared in a Pierce Spin column (ThermoFisher, 69705) containing 40 μl of Protein A/G PLUS‐Agarose (sc‐2003, Santa Cruz) for 1 h at 4°C on a rotor. Four Pierce Spin columns containing 40 μl of Protein A/G PLUS‐Agarose suspension were prepared. Three of the Pierce Spin columns were used to prepare the antibody/agarose complex and one of the columns was used as a negative control without containing the antibody. Five hundred microliters of HEPES buffer was added to each of the Protein A/G PLUS‐Agarose spin columns. For the Antibody/Agarose complex preparation, 10 μg of anti‐mouse CREB2 antibody (sc‐390063, Santa Cruz) was added to each of the Protein A/G PLUS‐Agarose Spin columns. The columns were incubated on a rotor at RT for 3 h. The columns were centrifuged at 2500 RPM for 1 min and the flow‐through was removed. The columns were then washed/centrifuged four times at 2500 RPM with HEPES buffer at 4°C. After the final wash, 500 μg of CNS homogenized lysate was transferred into each column containing the antibody/agarose complex and the one column without containing the antibody. The columns were placed on a rotor and incubated overnight at 4°C. The following day, the columns were centrifuged at 2500 RPM for 1 min and the lysate was removed. The columns were washed/centrifuged four times at 2500 RPM with HEPES buffer. The eluates for each column were eluted in 50 μl of Glycine, pH 2.8. The pH of the eluates was neutralized with 5 μl of 1 M Tris, pH 9.5. The IP products were pooled together and 100 μl of the IP product was used for LC/MS analysis and 45 μl of the IP product was prepared for western blotting. Forty‐five microliters of the IP product was resuspended in 15 μl of 4 × Loading sample buffer and boiled at 90°C for 10 min. Immunoprecipitates, negative control, and 25 μg of the pre‐immunoprecipitated lysate (5% input) were resolved in 10% SDS‐polyacrylamide gel and transferred to a nitrocellulose membrane. Non‐specific binding sites were blocked with 5% milk in TBS solution with 0.1% Tween‐20 (1× TBST) for 1 h at RT. Membranes were incubated with anti‐mouse CREB2 antibody (1:1000, Santa Cruz, sc‐390063) overnight at 4°C. The following day, the membrane was washed three times for 10 min in TBST and incubated with highly cross‐adsorbed Infra‐Red Dye 680RD goat anti‐mouse secondary antibody (1:5000, 926‐68070, LI‐COR) for 1 h at RT, followed by two washes with 1xTBST and a final wash of 1xPBS. Membranes were developed using the LI‐COR Odyssey Fc Fluorescence Imaging system.

#### 2.3.5 Protein precipitation for detergent removal

In order to remove the detergents used in our immunoprecipitation protocol, which are incompatible with the downstream MS analysis we have used the methanol/chloroform precipitation method as previously described.^34^ Protein precipitates were then resuspended in 50 μl of 5 X Invitrosol™ LC/MS Protein Solubilizer. The samples were incubated at 60°C for 5 min, vortexed, and incubated at 60°C for another 10 min. Two hundred microliters of 25 mM ammonium bicarbonate (ABC Buffer) was added per sample in order to obtain an Invitrosol™ concentration of 1X. The samples were separated into 5 tubes containing 50 μl of sample per tube.

#### 2.3.6 In‐solution Trypsin digestion with the MS detergents

Samples were reduced in 10 mM dithiothreitol (DTT) for 30 min at RT and cysteines were alkylated in the dark in 60 mM iodoacetamide (IAA) for 20 min at RT. Two microliters of Trypsin/Lys‐C mix (0.5 μg/μl, Promega, V5073) was added to each sample and incubated for 3 h at RT in the dark. After 3 h, 150 μl of ABC buffer per tube was added and incubated overnight at RT. The following day, the digestion was stopped by adding 200 μl of 5% acetonitrile and 3% trifluoroacetic acid. Tryptic peptides were desalted with stage tips as previously described.^35^ The desalted peptides were concentrated in a Speed‐Vac for 10 min at low power. The peptides were reconstituted with 15 μl of 5% acetonitrile, 0.1% formic acid, and transferred to a microplate for LC/MS analysis.

#### 2.3.7 LC/MS analysis

Peptides were separated by reverse‐phase chromatography using a Dionex Ultimate 3000 nanoLC (Dionex/Thermo Fisher Scientific) on a C18 capillary column (Acclaim PepMap100 C18, 2 μm, 100 Å, 75 μm i.d. × 20 cm/ThermoFisher) at an eluent flow rate of 300 nl/min. Mobile phase A contained 5% acetonitrile, 0.1% formic acid, and mobile phase B contained 80% acetonitrile and 0.1% formic acid. The column was preequilibrated with 5% mobile phase B followed by an increase to 65% mobile phase B in 75 min. The eluted peptides were ionized online by electrospray ionization (ESI) and transferred into an LTQ Orbitrap XL mass spectrometer (ThermoFisher) which was operated in the positive mode to measure full scan MS spectra (300–1700 m/z in the Orbitrap analyzer at resolution R = 30 000) followed by isolation and fragmentation (MS/MS) of the 10 most intense ions (in the LTQ iontrap) by collision‐induced dissociation (CID).

#### 2.3.8 MS/MS data analysis

The analysis of the raw MS and MS/MS Spectra was performed using the MaxQuant software (version. 1.6.3.4). Initial maximum precursor and fragment mass deviations were set to 7 ppm and 0.5 Da, respectively. Variable modification (oxidation of methionine and N‐terminal acetylation) and fixed modification (cysteine carbamidomethylation) were set for the search against a UniProt FASTA formatted database of the taxonomic clade *euthyneura* (including *Lymnaea stagnalis*). Trypsin with a maximum of two missed cleavages was chosen for digestion. The minimum peptide length was set to 7 amino acids and the false discovery rate (FDR) for peptide and protein identification was set to 0.01.

### 2.4 Recombinant protein of LymCBP

The partial recombinant protein of the LymCBP HAT domain (from 1240th arginine to 1641st lysine, GenBank accession number BAE02656.1) with His_6_‐tag was synthesized in *Escherichia coli* and purified as previously described, with slight modifications.^36^, ^37^, ^38^ LymCBP catalytic domain was amplified by RT‐PCR with cDNA prepared from *Lymnaea* CNSs, PrimeSTAR® HS DNA Polymerase (Takara Clontech), and primers, whose nucleotide sequences were summarized in Table S1 (No. 23, 24). To produce mutant LymCBP, PCR of overlap extension was performed using primer sets summarized in Table S1 (No. 25–32). The PCR product was ligated into pET28a(+) plasmid (Novagen), which was linearized with *Nhe* I and *Bam*H I, using the In‐Fusion® HD Cloning Plus kit (Takara Clontech). Then, the Rosetta‐gami™ 2(DE3) competent cells (Novagen) were transformed with this ligated plasmid. Expression of the partial recombinant protein of the LymCBP catalytic domain was induced by treatment with 0.32 mM of isopropyl β‐d‐thiogalactopyranoside (IPTG) in TBG‐M9 medium [0.8% Bacto Tryptone (BD) and 0.4% NaCl], followed by purification using nickel‐nitrilotriacetic acid (Ni‐NTA) agarose resin (QIAGEN). The recombinant protein was desalted with a PD‐10 column (Sephadex™ G‐25 M, GE Healthcare), purified using a cation exchange column [HiTrap carboxymethyl (CM) FF column (GE Healthcare)] installed in the ÄKTAprime plus system (GE Healthcare), and condensed with Amicon® Ultra‐15 column (Merck Millipore).

### 2.5 Acetylation assays using radioisotope‐labeled acetyl‐CoA

The procedures followed to perform this experiment were modified from previous reports.^36^, ^37^, ^38^, ^39^, ^40^ The recombinant LymCBP HAT domain (200 ng) was incubated with histone H1 purified from calf thymus (1 μg, Sigma‐Aldrich) and 7.4 kBq of [^14^C]‐acetyl‐CoA (Moravek, Inc.) for 4 h in buffer containing 50 mM Tris–HCl (pH 8.0), 10% glycerol, 1 mM dithiothreitol, and 10 mM sodium butyrate. Curcumin (MedChemExpress), anacardic acid (MedChemExpress), and garcinol (Enzo) prepared in dimethyl sulfoxide (DMSO) were premixed at 50 μM each. Reactions were separated in 14% SDS‐PAGE gels. To detect β radiation released from radiocarbon, the gels were dried on pieces of filter paper and exposed to imaging plates for several days. Signals were observed using a fluoro image analyzer (FLA‐2000; Fuji Film).

### 2.6 Intracellular recording

The whole CNS, including the cerebral ganglia and the buccal ganglia, was dissected from the snail in *Lymnaea* saline [50 mM NaCl, 1.6 mM KCl, 2.0 mM MgCl_2_, 3.5 mM CaCl_2_, and 10 mM HEPES (pH 7.9)]. The isolated CNS was immobilized on a Sylgard‐coated dish using stainless‐steel pins. In order to physically separate the cerebral giant cells and B1 motoneurons, a Vaseline dam was constructed around the cerebral ganglia. This enabled us to apply a solution that contains the inhibitors to the CGCs. The CGC and the B1 motoneuron were impaled with glass microelectrodes filled with 3 M KCl giving tip resistances of 20–50 MΩ. A train of 5 spikes in the CGC, which was produced by current injection (0.8–1.8 nA) for 1 s, evoked a single large compound EPSP in the B1 motoneuron (electric stimulator: SEN‐7203, Nihon Kohden, Tokyo, Japan; intracellular recording amplifier: MEZ‐8300, Nihon Kohden; AD converter: DIGIDATA 1322A, Axon Instruments, Foster City, CA, USA). We used the CGC and B1 neurons located in the ipsilateral cerebral and buccal ganglia, respectively.

### 2.7 Injection of cAMP or 5′AMP into the CGC

Based on our previous study,^41^ cyclic AMP (cAMP) or 5′AMP (Sigma‐Aldrich), was filled into a glass microelectrode (50–100 MΩ) as a 200 mM solution dissolved in 20 mM Tris buffer (pH 7.5), was injected into the CGC by passing hyperpolarizing current pulses (50 msec on, 50 msec off) of 4 nA for 20 min. Before and after the injection of hyperpolarizing current into the CGC, the EPSP recorded in the B1 motoneuron is not changed by activation of the CGC. The data were recorded in the same preparation at 30 min and 3 h after cAMP or 5′AMP injection. The data at 0 h (i.e., pre) were recorded before cAMP injection. For the analysis of EPSP changes recorded in the B1 motoneuron, the peak amplitude of the EPSP was measured.

### 2.8 Application of HAT inhibitors to the CGC

We used the following three different inhibitors of HATs: curcumin (Sigma), anacardic acid (Sigma), and garcinol (Enzo). Each of these drugs was dissolved in DMSO as a stock solution at 67.9 mM (curcumin), 50 mM (anacardic acid), and 20 mM (garcinol). Stock solutions were diluted 1000‐fold in *Lymnaea* saline and then applied to the CGC area where the cerebral ganglia were surrounded by a Vaseline wall and incubation for 1 h. After incubation with each drug, intracellular recordings for the measurement of EPSPs were made. The data were expressed as the mean ± SEM. Significant differences at *p* < .05 between pre‐injection, 30 min, and 3 h after injection cAMP or 5′AMP were examined by one‐way repeated measures ANOVA and post hoc Tukey's test. For the normalized EPSP amplitude, the values after 30 min and 3 h for each group compared to the pre‐injection were examined with one‐way repeated measured ANOVA and post hoc Tukey's test.

## 3 RESULTS

### 3.1 Properties of the LymCBP gene and protein

**FIGURE 1:**
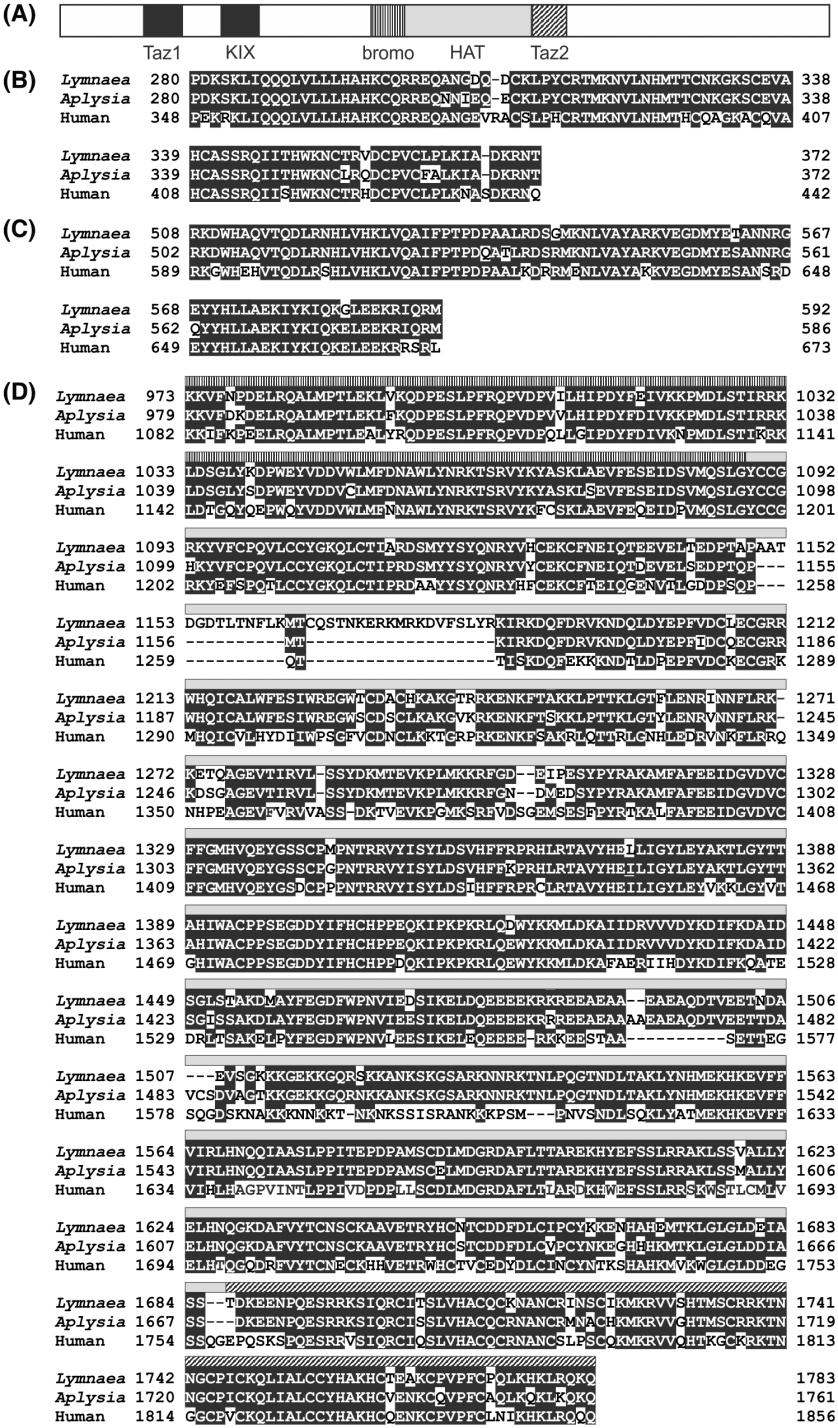
Predicted amino acid sequence of *Lymnaea* CBP. (A) Diagram of the structure of *Lymnaea* CBP (GenBank accession number: AB217914). Main functional domains (Taz1 domain, KIX domain, bromodomain, acetylation domain, and Taz2 domain) are highlighted with patterned bars. Alignments of amino acid sequences of (B) Taz1 domain, (C) KIX domain, and (D) compound of the bromodomain, HAT domain, and Taz2 domain between *Lymnaea* CBP and *Aplysia* CBP (GenBank accession number: AY064470) and human CBP (GenBank accession number: U47741). The patterns of bars in (D) correspond to those shown in (A).

The arrangement of the functional domains in LymCBP is the same as that of other p300/CBPs (Figure 1A). The full sequence of LymCBP (GenBank accession number: AB217914) in about 75% identical to *Aplysia californica* CBP (ApCBP; GenBank accession number: AAL54859) in the amino acid sequence level. In functional domains, such as Taz1 domain, KIX domain, bromodomain, HAT domain, and Taz2 domain of LymCBP, the amino acid sequences were individually over 90% identical to those of ApCBP and human CBP (GenBank accession number: U47741) (Figure 1B–D). Comparing amino acid sequences among LymCBP, human CBP, and human p300, the functional amino acid residues in the bromodomain for interaction with acetylated histones,^42^, ^43^, ^44^, ^45^ and those in the HAT domain for intramolecular interactions are highly conserved (Table S1).^46^, ^47^, ^48^, ^49^, ^50^ Molecular phylogenetic analysis of the p300/CBP proteins revealed that *Lymnaea* CBP is closely related to CBP proteins found in other molluscan species such as *Aplysia* and the oyster *Crassostrea giga* (Figure S1).

### 3.2 Localization of LymCBP mRNA in the *Lymnaea* CNS

**FIGURE 2:**
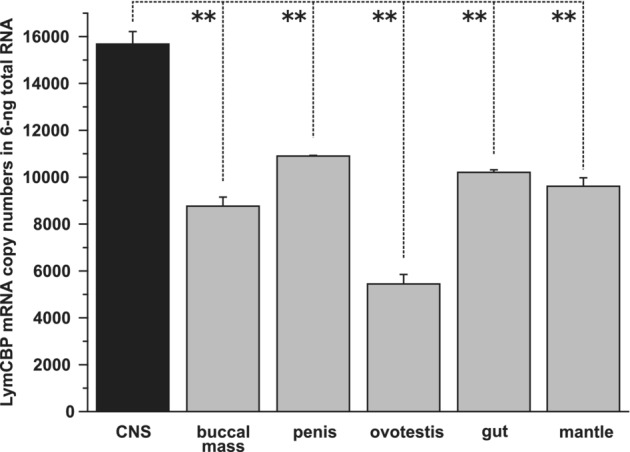
Quantification of LymCBP mRNA in the whole CNS of *Lymnaea*. LymCBP is more abundantly transcribed in the CNS than other tissues of *Lymnaea*. The data are expressed as the mean ± SEM; ANOVA, *p* < .01; **p < .01, Tukey's post hoc tests.

Absolute copy numbers of LymCBP mRNA in whole CNS and other tissues were analyzed by quantitative PCR (qPCR). More than 15 000 copies of LymCBP mRNA were contained in 6 ng of total RNA of CNSs (Figure 2). This value was 1.4 to 2.9 times higher compared to other tissues (Figure 2).

**FIGURE 3:**
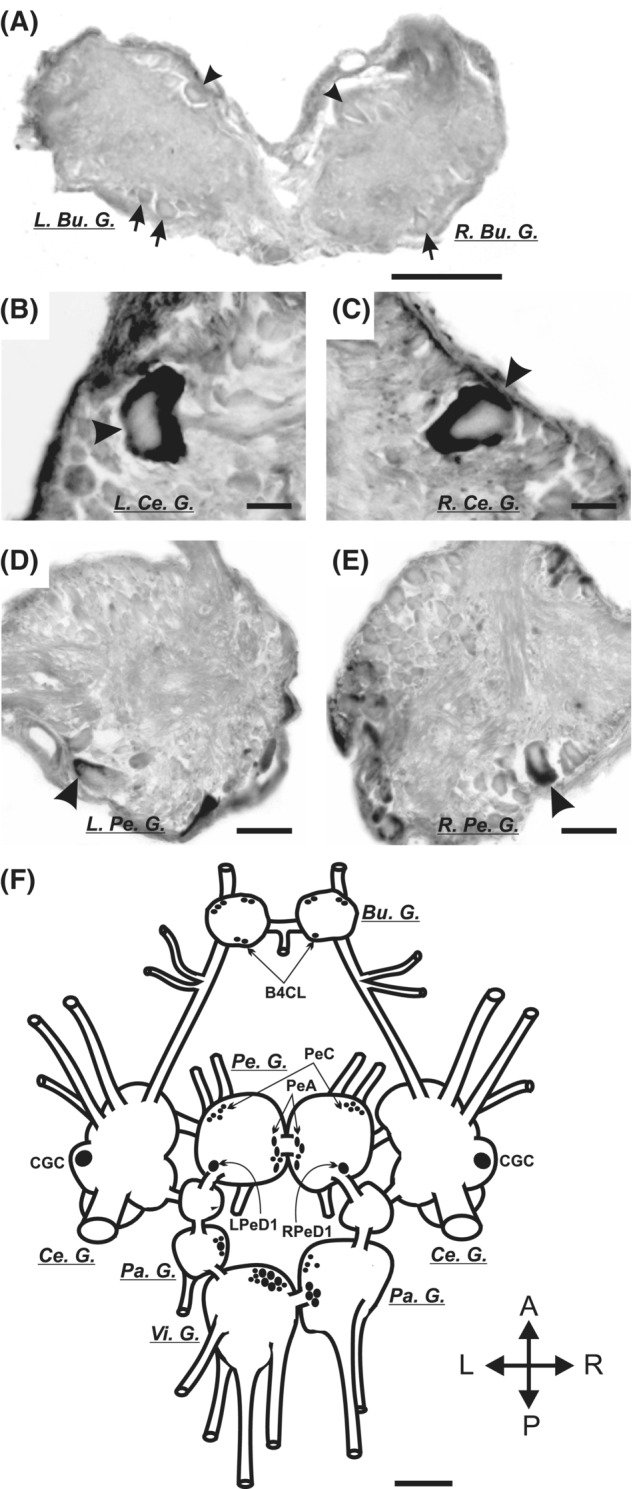
Localization of LymCBP mRNA in identifiable neurons shown by in situ hybridization. (A) Weak signals of LymCBP can be observed in several neurons (arrowheads), especially in the B4 cluster feeding motoneurons (arrows), in the buccal ganglia. Bu. G.: buccal ganglion, L: left; R: right. (B–E) Strong signal of LymCBP mRNA was observed in both CGCs (arrowheads in B and C), RPeD1 (arrowhead in D), and LPeD1 (arrowhead in E). Ce. G.: cerebral ganglion, Pe. G.: pedal ganglion. (F) Schematic drawing of the distribution of neurons containing LymCBP mRNA. Pa. G.: parietal ganglion, Vi. G.: visceral ganglion. Scale bars = 100 μm (A, D, and E) and 20 μm (B and C).

In situ hybridization showed the localization of LymCBP mRNA in the *Lymnaea* CNS samples (*n* = 8). While only a weak positive signal was detected in some neurons in the buccal ganglia (Figure 3A), a strong signal was observed in the CGCs in the cerebral ganglia (Figure 3B,C). The same signal was also seen in identifiable large neurons, the RPeD1 (Figure 3D) and LPeD1 (Figure 3E), in the pedal ganglia. Notably, the staining patterns of LymCBP mRNA in the buccal, cerebral, and pedal ganglia were very similar to those of LymCREB1.^24^ The same positive signal of LymCBP was also detected in unidentified neurons in the parietal and visceral ganglia (Figure 3F). No specific staining was detected in negative control experiments with the sense LymCBP mRNA probe (Figure S2).

### 3.3 Existence of the protein form of LymCBP

**FIGURE 4:**
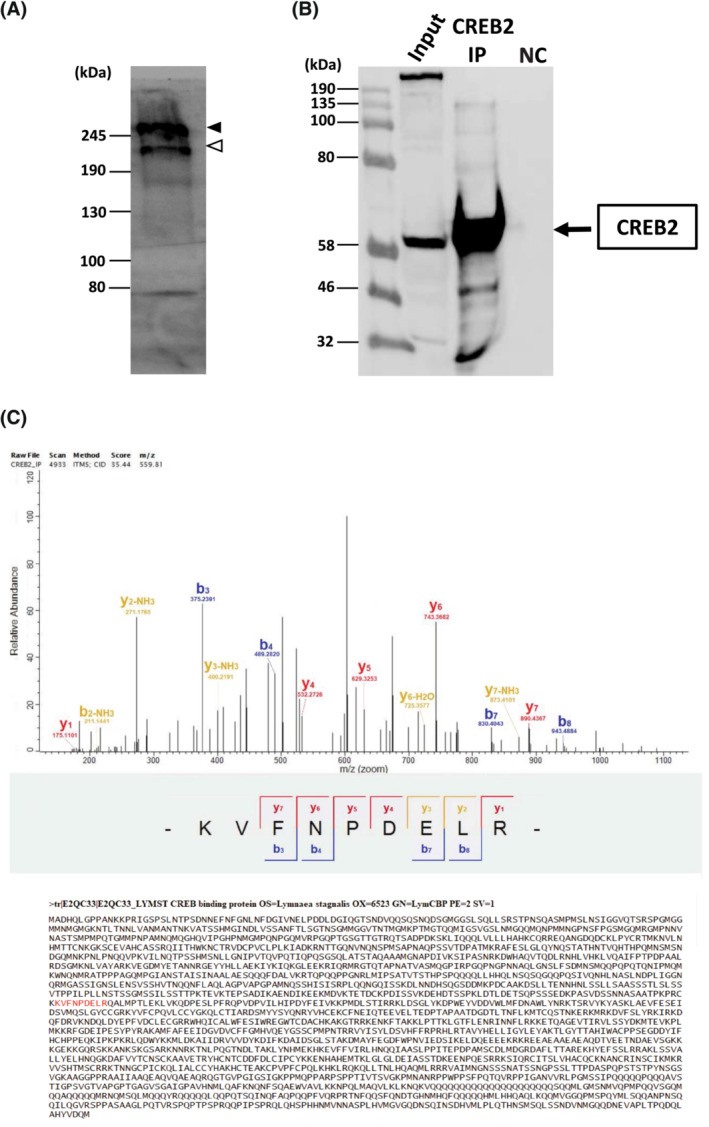
Identification of the protein form of LymCBP. (A) Existence of LymCBP protein in the *Lymnaea* CNS was confirmed by western blotting. The molecular weight of the positive band (245 kDa; filled arrowhead) was consistent with a theoretical value calculated from the amino acid sequence of LymCBP (252.3 kDa). A putative smaller isoform or post‐translationally cleaved form of LymCBP was also detected (open arrowhead). (B, C) CREB‐Binding Protein was identified as an interacting partner of CREB2 by MS/MS analysis in the CNS of *Lymnaea stagnalis*. (B) Sixty extracted CNSs of *Lymnaea stagnalis* were pooled, homogenized and subjected to immunoprecipitation using a specific CREB2 antibody. Immune complexes were immunoblotted for CREB2. (C) MS/MS Spectrum of the CBP identified as one of the interacting partners of CREB2 in the CNS of *Lymnaea stagnalis*. The CREB2 IP product shown in B was subjected to MS/MS analysis and a CBP‐specific peptide for *Lymnaea* CBP (KVFNPDELR) was identified. The x coordinate shows the peaks that come from both LymCREB2 and the proteins that bind to it specifically.

Western blotting demonstrated the existence of the protein form of LymCBP in the CNS. The epitope of the anti‐human‐CBP antibody is the *C*‐terminal region of human CBP, which is highly conserved in LymCBP (Figure 1D). The western blotting method detected two positive bands (Figure 4A). The larger band, indicated with a filled arrowhead around the marker of 245 kDa, was considered to be LymCBP, whose molecular weight was estimated as 252.3 kDa from the amino acid sequence (Figure 1). The smaller band indicated with an open arrowhead may be an isoform of LymCBP (Figure 4A). However, RT‐PCR techniques used for the molecular cloning of LymCBP and *in‐silico* searching using the transcriptome shotgun assembly sequence database of *Lymnaea* ^51^ could not find isoforms at the mRNA level. These results suggest that this putative smaller isoform of CBP is the result of some post‐translational modification, for example, cleaving by proteolysis.

Phosphorylation of CREB1 at Ser 133 is triggering its kinase inducible domain (KID) to bind to the KID interacting domain (KIX) of CREB binding protein (CBP).^52^, ^53^, ^54^ To identify the interaction of CBP with CREB proteins in our model organism *Lymnaea stagnalis*, we have utilized immunoprecipitation (IP) and liquid chromatography‐mass spectrometry (LC/MS). Although in pilot experiments we found that none of our CREB1 antibodies worked well for IP assays with total CNS lysates from *Lymnaea stagnalis*, previous studies have shown that human CREB2 (human activating transcription 4, hATF4), a transcriptional repressor of CREB1, can directly interact with multiple domains of the CBP. These domains include i. the KIX domain, ii. a region that contains the third zinc finger and the E1A‐interacting domain, iii. The *C*‐terminal region containing the p160/SRC‐1‐interacting domain and iii. The histone acetyltransferase domain can directly interact with the histone acetyltransferase p300 transcriptional coactivator of CBP.^55^ In addition, CBP and p300 can acetylate CREB2 in its bZIP domain and enhance its transcriptional activity.^55^, ^56^, ^57^ In order to confirm these reported interactions of CBP and CREB2, we have performed IP assays with a specific antibody for CREB2 and CNS homogenates from *Lymnaea stagnalis* (Figure 4B), followed by identification of the CREB2 interaction partners by LC/MS (Figure 4C). As shown in Figure 4C, after LC/MS, the data analysis of the CID fragmentation spectrum from a peptide with a mass of 559.81 m/z revealed the amino‐acid sequence of a unique peptide (KVFNPDELR) from the UniprotKB entry E2QC33 of *Lymnaea stagnalis*, validating that *Lymnaea* CBP is an interacting partner of CREB2 in the CNS. Importantly, these results further confirmed that LymCBP is expressed in the *Lymnaea* CNS.

### 3.4 Effects of HAT inhibitors on LymCBP enzymatic activity

In order to investigate the enzymatic properties of LymCBP as a postulated functional HAT, we first successfully produced a partial recombinant protein of its HAT domain (from 1240th to 1641st, Figures 1D and S3A,B). Next, to determine the optimal incubation temperature for the in vitro acetylation assay, a reaction mixture containing the recombinant LymCBP HAT domain protein, bovine histone H1, and [^14^C]‐acetyl‐CoA was incubated for 4 h at different temperatures; 15, 20, 25, 30, and 37°C. After separation by SDS‐PAGE, Coomassie Brilliant Blue (CBB) staining of the gel revealed the same amount of histone H1 at all five temperatures (lanes 1–5 in Figure S3C). However, the band of recombinant LymCBP HAT protein was almost completely absent after incubation at 37°C, suggesting that it was degraded at this higher temperature (lane 5 in Figure S3C).

In parallel autoradiography experiments, a positive signal indicating acetylation of histone H1 was observed when incubated at 15, 20, 25, and 30°C (lanes 6–9 in Figure S3C). Notably, similarly to the recombinant LymCBP HAT protein signal, the acetylated H1 signal was almost completely absent after incubation at 37°C (lane 10 in Figure S3C), indicating a likely dependence of H1 acetylation on the presence of a functional LymCBP HAT in these in vitro assays. Based on the outcome of these experiments, we incubated samples at 30°C in all subsequent assays.

Having established that H1 acetylation occurs in the presence of the recombinant LymCBP HAT protein, we set out to test if this acetylation is blocked by known HAT inhibitors, namely curcumin, anacardic acid, and garcinol. As the first step in these assays, the effect of the HAT inhibitors on total H1 levels was investigated. After adding each HAT inhibitor at 50 μM final concentration, or DMSO as a negative control, samples were incubated for 4 h at 30°C and separated by SDS‐PAGE. CBB staining revealed the presence of histone H1 after incubation with all three inhibitors (lanes 11 to 14 in Figure S3D), as well as without incubation with them (no HATi), indicating that the inhibitors do not cause the degradation of the H1 protein.

**FIGURE 5:**
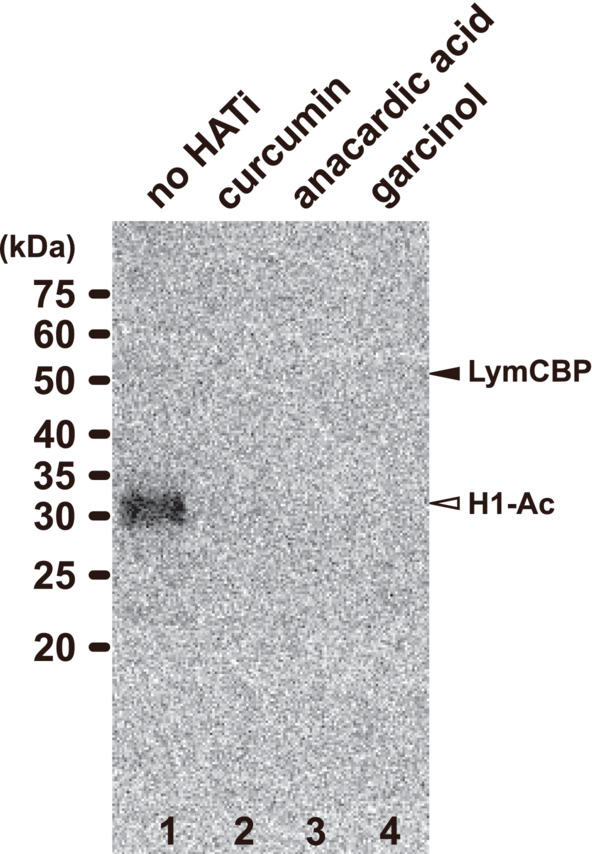
HAT activity of LymCBP is blocked by inhibitors. Effects of the HAT inhibitors curcumin, anacardic acid, and garcinol were tested with autoradiography. Reaction mixtures were incubated at 30°C. Without any HAT inhibitor (no HATi), a strong positive signal of acetylation was detected in the band of histone H1 (lane 1, H1‐Ac). This signal was eliminated by the addition of each HAT inhibitor (lanes 2–4), showing that the acetylation activity of the LymCBP recombinant protein was significantly blocked by HAT inhibitors.

Finally, we tested the ability of curcumin, anacardic acid, and garcinol to block the ability of the LymCBP HAT protein to acetylate histone H1. Autoradiography showed that in the absence of HAT inhibitors in the incubation solution, LymCBP acetylated histone H1 (lane 1 in Figure 5). In the presence of HAT inhibitors in the incubation solution, however, H1 acetylation signals were eliminated (lanes 2–4 in Figure 5), indicating that LymCBP HAT activity was blocked. These results showed that similar to human CBP, LymCBP has HAT activity which is involved in histone acetylation, and this enzymatic activity is significantly blocked by HAT inhibitors.

We attempted to produce mutant recombinant proteins of the LymCBP HAT domain and investigate alterations of HAT activity resulting from these mutations. We selected 6 single/double amino‐acid mutations, which had been reported to decrease the HAT activity of mammalian CBPs (Figure S4A).^9^, ^12^, ^58^ These amino acid residues were also conserved in LymCBP (Figures S4A and S5A). However, the expression levels of all 6 mutant proteins were lower than that of the wild‐type recombinant proteins (Figures S4B and S5B). Moreover, after the treatment of desalting, the amounts of these mutant proteins were further decreased and proved insufficient for purification by cation‐exchange chromatography (Figures S4C and S5C), preventing the testing of their acetyltransferase activity.

### 3.5 Synaptic plasticity between a buccal motoneuron and its regulatory interneuron induced by cAMP

**FIGURE 6:**
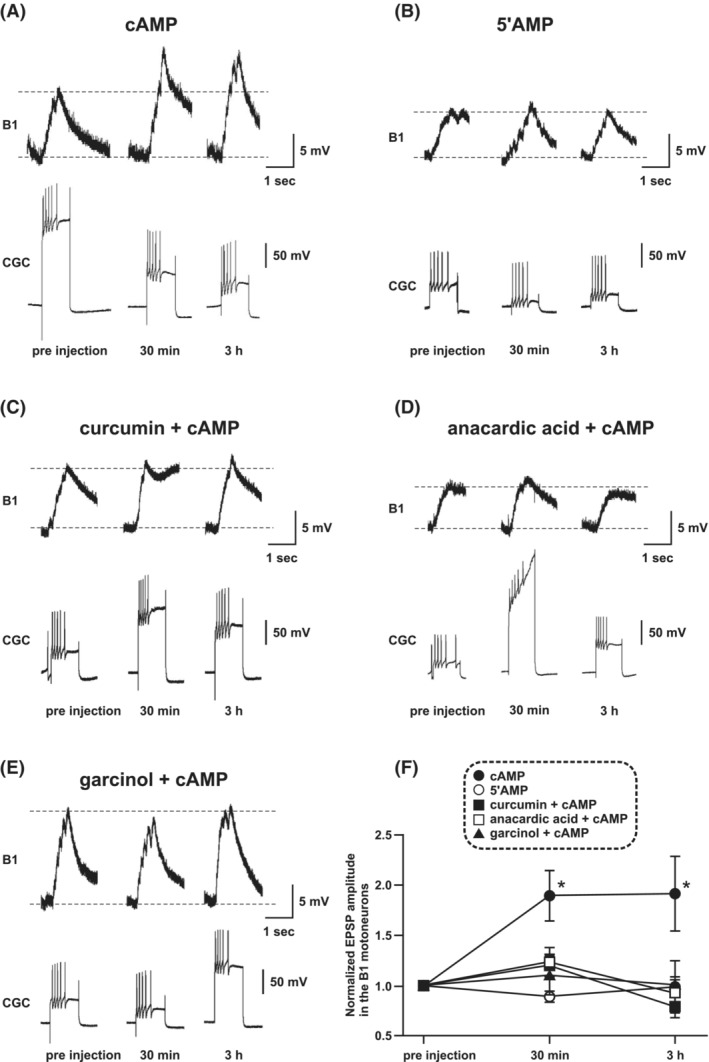
cAMP‐induced facilitation of the CGC to B1 motoneuron synapse is blocked by HAT inhibitors. EPSPs were evoked in the B1 motoneuron by the injection of depolarizing current into the CGC. The isolated CNS was incubated with DMSO saline (vehicle) or HAT inhibitors for 1 h prior to injection of cAMP. (A) cAMP injection into the CGC cell body increased EPSP amplitude in the B1 motoneuron at both 30 min and 3 h after the injection. (B) In contrast, EPSP amplitude increase was not observed in the group which was injected with 5′AMP. (C, D, E) The CNS was incubated with saline containing one of the HAT inhibitors curcumin (67.9 μM), anacardic acid (50 μM), or garcinol (20 μM) for 1 h prior to injection of the cAMP. In these groups, the EPSP was not increased either at 30 min or 3 h after the injection of cAMP. (F) The enhancement of B1 EPSPs is shown in the summarized data. Injection of cAMP into the CGC cell body increased EPSP amplitude in the B1 motoneuron, but pre‐treatment with either HAT inhibitor did not elicit enlargement of EPSP. The data are expressed as the mean ± SEM, *p < .05, Tukey's post hoc test.

The isolated *Lymnaea* CNS was incubated with DMSO saline for 1 h before the injection of cAMP into the CGC cell body. We tested the changes in the EPSP in the B1 motoneuron by activation of the CGC after the cAMP injection. We measured the amplitudes of the compound EPSPs 30 min and 3 h after the onset of cAMP injection. The CGC was depolarized by current injection to fire action potentials, evoking the compound EPSP in the B1 motoneuron. We used a train of 5 spikes in the CGC to evoke large compound EPSPs in the B1 motoneuron because the single EPSPs in the B1 motoneuron were very small (approximately 1 mV). These compound EPSPs at 30 min and 3 h after in preparations with cAMP‐injected CGCs were about twice as large as pre‐injection EPSPs (Figure 6A, *F* (2, 18) = 10.35, pre‐injection vs 30 min: *p* < .05; pre‐injection vs 3 h: *p* < .05, *n* = 10). In contrast, there were no significant changes in the 5′AMP‐injected preparations (Figure 6B, *F* (2, 12) = 1.192, pre‐injection vs 30 min: *p* > .05; pre‐injection vs 3 h: *p* > .05, *n* = 7). Thus, the effect of HAT inhibitors on the compound EPSPs 30 min and 3 h after cAMP injection were examined in the following experiments.

### 3.6 Effects of HAT inhibitors on the EPSP in the B1 motoneuron

As shown in Figure 5, the HAT inhibitors curcumin, anacardic acid, and garcinol possess significant inhibitory effects on the HAT activity of LymCBP. Therefore, we examined the effects of the application of these HAT inhibitors to CGCs on the compound B1 EPSPs that were found to be increased after cAMP injection into the CGC. As reported previously,^24^, ^41^ the EPSP in the B1 motoneuron is facilitated by injection of cAMP into the CGC (Figure 6A). This increase in EPSP amplitude was not observed when 5′AMP was injected into CGCs instead of cAMP (Figure 6B).

Next, we investigated changes to the cAMP‐induced EPSP in the presence of curcumin, anacardic acid, and garcinol, respectively. After incubation with 67.9 μM curcumin of the cerebral ganglion for 1 h, cAMP injection into the CGC did not result in a significant increase of the amplitude of the compound EPSPs in the B1 motoneuron, either at 30 min or 3 h after cAMP injection, compared to pre‐injection (Figure 6C, *F* [2, 22] = 6.001, pre‐injection vs 30 min: *p* > .05; pre‐injection vs 3 h: *p* > .05, *n* = 12). Similarly, incubation with 50 μM anacardic acid of the cerebral ganglion for 1 h prevented the cAMP‐induced facilitation of the compound EPSPs in the B1 motoneuron (Figure 6D, *F* [2, 10] = 1.563, pre‐injection vs 30 min: *p* > .05; pre‐injection vs 3 h: *p* > .05, *n* = 6). Finally, incubation with 20 μM garcinol of the cerebral ganglion for 1 h also prevented the cAMP‐induced facilitation of the compound EPSPs in the B1 motoneuron (Figure 6E, *F* [2, 14] = 0.01214, pre‐injection vs 30 min: *p* > .05; pre‐injection vs 3 h: *p* > .05, *n* = 8).

The results from all these groups are summarized in Figure 6F. The effect of HAT inhibitors on increasing compound EPSPs amplitude was tested by taking a 0 min baseline recording followed by a 30 min and a 3 h recording after cAMP was injected. The compound EPSPs were normalized to the initial baseline value. Incubation with either of the three HAT inhibitors before the injection of cAMP prevented the facilitation of compound EPSPs. In the post hoc Tukey's test, there were significant differences in the following comparisons: DMSO saline treatment cAMP injection group; pre‐injection vs 30 min; pre‐injection vs 3 h, curcumin treatment group; 30 min vs 3 h.

## 4 DISCUSSION

In the present study we, for the first time, identified a conserved CREB‐binding protein in the CNS of *Lymnaea* (LymCBP) and demonstrated its HAT activity in biochemical assays. We also showed the importance of HAT activity for cAMP‐induced synaptic plasticity between an identified interneuron (CGC) and a postsynaptic motoneuron (B1) of the feeding system. The CGC is a key interneuron for consolidating both aversive and appetitive LTM for taste, and both LymCBP and LymCREB1 are transcribed in this neuron (Figure 3). A previous report showed that inhibiting functions of LymCREB1 by pre‐injection of excess amount of a CRE oligonucleotide and siRNA of LymCREB1 into the CGC blocked cAMP‐induced synaptic facilitation between the CGCs and the B1 motoneurons,^24^, ^29^ and suggested a crucial role of LymCREB1 as a transcriptional factor in synaptic facilitation. Here, the inhibition of HAT activity using HAT inhibitors similarly blocked cAMP‐induced synaptic facilitation (Figure 6). The biochemical detection of LymCBP HAT activity supports the notion that this synaptic facilitation requires the cooperation of LymCREB1 and LymCBP.

### 4.1 Gene structure of LymCBP

We succeeded in cloning the full‐length cDNA of LymCBP from the *Lymnaea* CNS. Comparing its deduced amino acid sequence with those of *Aplysia* and human CBPs, several functional domains were found to be highly conserved (Figure 1). This result suggests that LymCBP has similar functions to mammalian CBP and p300 (Figure S1). The KIX domain of CBP associates with the kinase‐inducible domain (KID) of CREB, and they construct a complex together.^59^ KID, which is also called as P‐box, is highly conserved in LymCREB1.^24^ High conservation of the KIX domain in LymCBP and KID in LymCREB1 suggested that LymCBP and LymCREB could interact via these domains. Additionally, a previous report showed that the KIX domain of CBP is involved in the formation of spatial memory following hippocampus‐dependent learning.^60^ Previous studies^24^, ^26^, ^29^ and our present work (Figure 3) indicated that these two mRNAs are co‐localized in the CGCs, key neurons for LTM consolidation. Taken together, the consolidation of LTM in *Lymnaea* is suggested to be regulated by LymCBP and LymCREB1.

The Taz1 and Taz2 domains of LymCBP were also highly conserved (Figure 1). These domains function as interfaces to bind with a variety of transcription factors and HATs, such as Fos and p300/CBP‐associated factor (pCAF).^61^ It was previously reported that the bromodomain of mammalian CBP is important for the acetylation of histones^60^ and interaction with acetylated histones.^44^, ^61^ At least 15 amino acid residues in the bromodomain regulate interaction with acetylated histones,^42^, ^44^, ^45^ and, compared with amino acid sequences among human p300, human CBP, and LymCBP, 14 out of 15 amino acid residues were conserved in LymCBP (Table S3). Other functional amino acid residues are highly conserved in the RING domain (Table S4), the acetyl‐CoA‐binding domain (Table S5), and the ZZ domain (Table S6). The RING domain in human p300/CBP has an autoinhibitory function for the HAT domain.^46^, ^47^ The ZZ domain of human p300/CBP recognizes histone tails and modulates its enzymatic activity.^48^, ^62^ The most important domain in CBP is the KIX domain, which is well known as an interface for interacting with CREB,^63^ and this domain is highly conserved in LymCBP (Figure 1). These results suggested that not only the HAT activity, which was shown in Figure 5, but also other functions of CBP are highly conserved in LymCBP.

Our immunoprecipitation assays indicated an interaction between LymCBP and LymCREB2 (Figure 4B). A similar interaction also has been shown in the human homologs of these proteins.^55^ The interfaces in CBP for binding with CREB2 include the KIX domain, Taz2 domain, and HAT domain.^55^ These domains are highly conserved in LymCBP (Figure 1). In addition, acetylation of CREB2 by CBP was previously reported.^56^ Therefore, the interaction between LymCBP and LymCREB2 shown in Figure 4B may be needed for LymCREB2 acetylation by LymCBP.

### 4.2 Expression of LymCBP in the *Lymnaea* CNSs

In situ hybridization showed that LymCBP mRNA was transcribed in a limited number of identifiable neurons (Figure 3F). In particular, the CGCs in the cerebral ganglia exhibited a strong LymCBP signal (Figure 3B,C). Both mRNA and protein forms of LymCREB1 exist in the CGC,^24^, ^26^ showing the co‐localization of LymCBP and LymCREB1 in this neuron. The CGC was identified to be a key neuron for the long‐lasting conditioned responses after taste aversion learning^27^, ^64^, ^65^, ^66^ and appetitive classical conditioning.^18^, ^28^, ^67^, ^68^, ^69^ The same signal was also seen in identified large neurons, the RPeD1, and the LPeD1, in the pedal ganglia (Figure 3D,E). The RPeD1 was especially reported to be necessary for LTM formation in operant conditioning of aerial respiratory behavior.^70^ The staining patterns of LymCBP mRNA in the buccal, cerebral, and pedal ganglia were very similar to those of LymCREB1, which possesses KID for interacting with LymCBP and plays a role in the enhancement of transcriptional activity.^24^ Our proteomics experiments confirmed the existence of translated LymCBP in the *Lymnaea* CNS, corroborating the findings from the DNA and RNA level assays of LymCBP expression.

In addition, we previously demonstrated the existence of both mRNA and protein forms of another transcriptional factor, CCAAT/enhancer binding protein of *Lymnaea* (LymC/EBP), in the CGC and the RPeD1.^71^ In mammalian cells, C/EBP is acetylated by p300/CBP, its acetylation boosts C/EBP‐mediated transcriptional activity.^72^ Taken together, the interaction between LymCBP and transcriptional factors, such as LymCREB1, LymCREB2, and LymC/EBP, may be involved in the consolidation of LTM of *Lymnaea*.

### 4.3 Involvement of LymCBP HAT activity in synaptic plasticity

In this study, we used HAT inhibitors (curcumin, anacardic acid, and garcinol) for biochemical and electrophysiological experiments (Figures 5 and 6).^73^ Biochemical analyses using the recombinant LymCBP HAT domain showed that the HAT activity of LymCBP was significantly blocked by HAT inhibitors. Molecular docking simulation indicated the amino acid residues of the human p300, whose amino acid sequence of the HAT domain was highly identical with human CBP, for interaction with garcinol.^74^ For interaction between human p300 and garcinol, five amino acid residues (S1400, Y1414, D1444, Y1446, and Q1455) in its HAT domain were required, and these were completely conserved in LymCBP (S1356, Y1370, D1400, Y1402, and Q1411) (Table S7). Therefore, the HAT activity of LymCBP might be inhibited by similar interaction mechanisms with garcinol. We failed in our attempt to produce sufficient quantities of mutant recombinant proteins of the LymCBP HAT domain (Figure S4) to allow us to test the alteration of the HAT activity after amino acid substitution. However, many mutations affecting the five amino acid residues that are highly conserved in LymCBP have been reported to cause a decrease in HAT activity^9^, ^12^, ^58^ so with the right conditions for expression and purification, it may be possible to confirm in future work that this is also the case in the *Lymnaea* CNS.

Our electrophysiological experiments showed that the HAT inhibitors used in our biochemical experiments inhibited cAMP‐dependent synaptic plasticity between the CGCs and their follower neurons (Figure 6). Although the block of synaptic plasticity by curcumin has not been reported in other species, its inhibitory effects on newly acquired or reactivated fear memories were indicated.^75^ Similarly, garcinol was also reported to block newly acquired memories in mammals and honeybees.^14^, ^16^, ^17^, ^75^ Another HAT inhibitor, C646, which was not used in this study, significantly impaired memory consolidation.^16^, ^76^ These inhibitory effects might be induced by blocking the HAT activities of CBP and other HATs. On the contrary, there are many reports showing that curcumin has ameliorative effects on the impairment of memory and synaptic plasticity. Curcumin is a multi‐target agent and might interact with various target molecules that have a positive effect on memory. Especially, curcumin is widely used as an agent for Alzheimer's disease, and its anti‐amyloid efficacy already has been reported.^77^, ^78^ For recovering from chronic unpredictable stress‐induced cognitive deficits, curcumin effectively inhibited the reduction of hippocampal BDNF and ERK levels.^79^ Curcumin was also effective in scopolamine‐induced memory retrieval deficit by preventing reduction of Akt and GSK‐3β phosphorylation in the hippocampus.^79^ In addition, curcumin has been reported to ameliorate memory consolidation deficits caused by heavy metals,^80^ aging,^81^, ^82^ and viral proteins.^83^, ^84^ All these effects of curcumin are based on its role in consolidating or already consolidated long‐term memory but this does not rule out that its effect is inhibitory in the earliest stage of memory consolidation when histone acetylation plays a key role in the formation of memory.

## AUTHOR CONTRIBUTIONS

Experiments performed in the UK: D. Hatakeyama., I. Felletár, A. Anagnostopoulou, M. Eravci, I. Kemenes, and G. Kemenes conceived and designed the experiments. I. Felletár and A. Fitchett performed the western blot assays. A. Anagnostopoulou and M. Eravci performed the immunoprecipitation and LC–MS experiments, respectively. I. Felletár, A. Fitchett, A. Anagnostopoulou, M. Eravci, and G. Kemenes analyzed and visualized the data. A. Anagnostopoulou, M. Eravci, I. Kemenes, and G. Kemenes wrote the relevant results sections of the paper. G. Kemenes and I. Kemenes obtained the funding for the work performed at the University of Sussex.

Experiments performed in Japan: D. Hatakeyama conceived and designed the experiments. D. Hatakeyama and Y. Totani purified total RNA from *Lymnaea* tissues. D. Hatakeyama, R. Miki, A. Okada, N. Abe, and T. Kuzuhara prepared the LymCBP recombinant proteins and performed acetylation assay. H. Sunada and E. Ito performed electrophysiological experiments. T. Watanabe created the phylogenetic tree.

D. Hatakeyama wrote the first version of the paper and all the authors contributed to the final version.

## FUNDING INFORMATION

This work was partly supported by Waseda University Grants for Special Research Projects (2018 K‐141 and 2020C‐135) to E.I. Work at the University of Sussex, UK, was funded by grants from the Medical Research Council to G.K. (MRC/G0400551), and the Biotechnology and Biological Sciences Research Council (BBSRC) to G.K. and I.K. (BB/H009906/1) and I.K. and G.K. (BB/P00766X/1), respectively.

## ACKNOWLEDGEMENTS

We are grateful to the organizations mentioned in the Funding information for their financial support of our work.

## DISCLOSURES

The authors declare no competing financial interests.

## Supporting information


Figure S1

## Data Availability

All data needed to evaluate the conclusions of the study are presented in the paper and/or the Supporting Information. Detailed numerical data are available on FigShare (https://doi.org/10.25377/sussex.21263289: [TBA]). Additional data related to this paper may be requested from the authors.

## References

[1] Kandel ER . The molecular biology of memory storage: a dialogue between genes and synapses. Science. 2001;294:1030‐1038. 11691980 10.1126/science.1067020

[2] Matos MR , Visser E , Kramvis I , et al. Memory strength gates the involvement of a CREB‐dependent cortical fear engram in remote memory. Nat Commun. 2019;10:2315. 31127098 10.1038/s41467-019-10266-1 PMC6534583

[3] Laviv T , Scholl B , Parra‐Bueno P , et al. In vivo imaging of the coupling between neuronal and CREB activity in the mouse brain. Neuron. 2020;105:799‐812. 31883788 10.1016/j.neuron.2019.11.028 PMC7144870

[4] Lakhina V , Arey RN , Kaletsky R , et al. Genome‐wide functional analysis of CREB/long‐term memory‐dependent transcription reveals distinct basal and memory gene expression programs. Neuron. 2015;85:330‐345. 25611510 10.1016/j.neuron.2014.12.029 PMC4340687

[5] Zhou L , Zhang Y , Liu RY , Smolen P , Cleary LJ , Byrne JH . Rescue of impaired long‐term facilitation at sensorimotor synapses of *Aplysia* following siRNA knockdown of CREB1. J Neurosci. 2015;35:1617‐1626. 25632137 10.1523/JNEUROSCI.3330-14.2015 PMC4308605

[6] Hirano Y , Ihara K , Masuda T , et al. Shifting transcriptional machinery is required for long‐term memory maintenance and modification in *Drosophila* mushroom bodies. Nat Commun. 2016;7:13471. 27841260 10.1038/ncomms13471 PMC5114576

[7] Cedar H , Bergman Y . Linking DNA methylation and histone modification: patterns and paradigms. Nat Rev Genet. 2009;10:295‐304. 19308066 10.1038/nrg2540

[8] Alarcón JM , Malleret G , Touzani K , et al. Chromatin acetylation, memory, and LTP are impaired in CBP^+/−^ mice: a model for the cognitive deficit in Rubinstein‐Taybi syndrome and its amelioration. Neuron. 2004;42:947‐959. 15207239 10.1016/j.neuron.2004.05.021

[9] Korzus E , Rosenfeld MG , Mayford M . CBP histone acetyltransferase activity is a critical component of memory consolidation. Neuron. 2004;42:961‐972. 15207240 10.1016/j.neuron.2004.06.002 PMC8048715

[10] Chatterjee S , Mizar P , Cassel R , et al. A novel activator of CBP/p300 acetyltransferases promotes neurogenesis and extends memory duration in adult mice. J Neurosci. 2013;33:10698‐10712. 23804093 10.1523/JNEUROSCI.5772-12.2013 PMC6618502

[11] Guan Z , Giustetto M , Lomvardas S , et al. Integration of long‐term‐memory‐related synaptic plasticity involves bidirectional regulation of gene expression and chromatin structure. Cell. 2002;111:483‐493. 12437922 10.1016/s0092-8674(02)01074-7

[12] Martinez‐Balbás MA , Bannister AJ , Martin K , Haus‐Seuffert P , Meisterernst M , Kouzarides T . The acetyltransferase activity of CBP stimulates transcription. EMBO J. 1998;17:2886‐2893. 9582282 10.1093/emboj/17.10.2886 PMC1170629

[13] Vecsey CG , Hawk JD , Lattal KM , et al. Histone deacetylase inhibitors enhance memory and synaptic plasticity via CREB:CBP‐dependent transcriptional activation. J Neurosci. 2007;27:6128‐6140. 17553985 10.1523/JNEUROSCI.0296-07.2007 PMC2925045

[14] Zhao Z , Fan L , Fortress AM , Boulware MI , Frick KM . Hippocampal histone acetylation regulates object recognition and the estradiol‐induced enhancement of object recognition. J Neurosci. 2012;32:2344‐2351. 22396409 10.1523/JNEUROSCI.5819-11.2012 PMC3401048

[15] Monsey MS , Gerhard DM , Boyle LM , Briones MA , Seligsohn M , Schafe GE . A diet enriched with curcumin impairs newly acquired and reactivated fear memories. Neuropsychopharmacology. 2015;40:1278‐1288. 25430781 10.1038/npp.2014.315 PMC4367473

[16] Merschbaecher K , Hatko L , Folz J , Mueller U . Inhibition of different histone acetyltransferases (HATs) uncovers transcription‐dependent and ‐independent acetylation‐mediated mechanisms in memory formation. Learn Mem. 2016;23:83‐89. 26773101 10.1101/lm.039438.115 PMC4749833

[17] Maddox SA , Watts CS , Doyère V , Schafe GE . A naturally‐occurring histone acetyltransferase inhibitor derived from *Garcinia indica* impairs newly acquired and reactivated fear memories. PLoS One. 2013;8:e54463. 23349897 10.1371/journal.pone.0054463 PMC3549978

[18] Kemenes I , Straub VA , Nikitin ES , et al. Role of delayed nonsynaptic neuronal plasticity in long‐term associative memory. Curr Biol. 2006;16:1269‐1279. 16824916 10.1016/j.cub.2006.05.049

[19] Hatakeyama D , Okuta A , Otsuka E , Lukowiak K , Ito E . Consolidation of long‐term memory by insulin in *Lymnaea* is not brought about by changing the number of insulin receptors. Commun Integr Biol. 2013;6:e23955. 23710281 10.4161/cib.23955 PMC3656023

[20] Murakami J , Okada R , Sadamoto H , et al. Involvement of insulin‐like peptide in long‐term synaptic plasticity and long‐term memory of the pond snail *Lymnaea stagnalis* . J Neurosci. 2013;33:371‐383. 23283349 10.1523/JNEUROSCI.0679-12.2013 PMC6618621

[21] Totani Y , Nakai J , Dyakonova VE , Lukowiak K , Sakakibara M , Ito E . Induction of LTM following an insulin injection. eNeuro. 2020;7: ENEURO.0088‐20.2020. 10.1523/ENEURO.0088-20.2020 PMC7218004 32291265

[22] Nakai J , Totani Y , Hatakeyama D , Dyakonova VE , Ito E . Another example of conditioned taste aversion: case of snails. Biology (Basel). 2020;9:422. 33256267 10.3390/biology9120422 PMC7760351

[23] Nakai J , Totani Y , Kojima S , Sakakibara M , Ito E . Features of behavioral changes underlying conditioned taste aversion in the pond snail *Lymnaea stagnalis* . Invert Neurosci. 2020;20:8. 32385589 10.1007/s10158-020-00241-7

[24] Sadamoto H , Sato H , Kobayashi S , et al. CREB in the pond snail *Lymnaea stagnalis*: cloning, gene expression, and function in identifiable neurons of the central nervous system. J Neurobiol. 2004;58:455‐466. 14978723 10.1002/neu.10296

[25] Sadamoto H , Kitahashi T , Fujito Y , Ito E . Learning‐dependent gene expression of CREB1 isoforms in the molluscan brain. Front Behav Neurosci. 2010;4:25. 20631825 10.3389/fnbeh.2010.00025 PMC2901150

[26] Ribeiro MJ , Serfőző Z , Papp A , et al. Cyclic AMP response element‐binding (CREB)‐like proteins in a molluscan brain: cellular localization and learning‐induced phosphorylation. Eur J Neurosci. 2003;18:1223‐1234. 12956721 10.1046/j.1460-9568.2003.02856.x

[27] Ito E , Otsuka E , Hama N , et al. Memory trace in feeding neural circuitry underlying conditioned taste aversion in *Lymnaea* . PLoS One. 2012;7:e43151. 22900097 10.1371/journal.pone.0043151 PMC3416747

[28] Nikitin ES , Balaban PM , Kemenes G . Nonsynaptic plasticity underlies a compartmentalized increase in synaptic efficacy after classical conditioning. Curr Biol. 2013;23:614‐619. 23541730 10.1016/j.cub.2013.02.048

[29] Wagatsuma A , Azami S , Sakura M , Hatakeyama D , Aonuma H , Ito E . *De novo* synthesis of CREB in a presynaptic neuron is required for synaptic enhancement involved in memory consolidation. J Neurosci Res. 2006;84:954‐960. 16886187 10.1002/jnr.21012

[30] Hatakeyama D , Sadamoto H , Ito E . Real‐time quantitative RT‐PCR method for estimation of mRNA level of CCAAT/enhancer binding protein in the central nervous system of *Lymnaea stagnalis* . Acta Biol Hung. 2004;55:157‐161. 15270230 10.1556/ABiol.55.2004.1-4.19

[31] Hatakeyama D , Sadamoto H , Watanabe T , et al. Requirement of new protein synthesis of a transcription factor for memory consolidation: paradoxical changes in mRNA and protein levels of C/EBP. J Mol Biol. 2006;356:569‐577. 16403525 10.1016/j.jmb.2005.12.009

[32] Wagatsuma A , Sadamoto H , Kitahashi T , Lukowiak K , Urano A , Ito E . Determination of the exact copy numbers of particular mRNAs in a single cell by quantitative real‐time RT‐PCR. J Exp Biol. 2005;208:2389‐2398. 15939778 10.1242/jeb.01625

[33] Hatakeyama D , Mita K , Kobayashi S , et al. Glutamate transporters in the central nervous system of a pond snail. J Neurosci Res. 2010;88:1374‐1386. 19937812 10.1002/jnr.22296

[34] Wessel D , Flugge UI . A method for the quantitative recovery of protein in dilute solution in the presence of detergents and lipids. Anal Biochem. 1984;138:1417‐1143. 10.1016/0003-2697(84)90782-6 6731838

[35] Rappsilber J , Ishihama Y , Mann M . Stop and go extraction tips for matrix‐assisted laser desorption/ionization, nanoelectrospray, and LC/MS sample pretreatment in proteomics. Anal Chem. 2003;75:663‐670. 12585499 10.1021/ac026117i

[36] Hatakeyama D , Shoji M , Yamayoshi S , et al. A novel functional site in the PB2 subunit of influenza a virus essential for acetyl‐CoA interaction, RNA polymerase activity, and viral replication. J Biol Chem. 2014;289:24980‐24994. 25063805 10.1074/jbc.M114.559708 PMC4155666

[37] Hatakeyama D , Shoji M , Yamayoshi S , et al. Influenza a virus nucleoprotein is acetylated by histone acetyltransferases, PCAF and GCN5. J Biol Chem. 2018;293:7126‐7138. 29555684 10.1074/jbc.RA117.001683 PMC5950015

[38] Hatakeyama D , Shoji M , Ogata S , et al. Acetylation of the influenza a virus polymerase subunit PA in the N‐terminal domain positively regulates its endonuclease activity. FEBS J. 2022;289:231‐245. 34270849 10.1111/febs.16123

[39] Hatakeyama D , Ohmi N , Saitoh A , et al. Acetylation of lysine residues in the recombinant nucleoprotein and VP40 matrix protein of Zaire ebolavirus by eukaryotic histone acetyltransferases. Biochem Biophys Res Comm. 2018;504:635‐640. 30205953 10.1016/j.bbrc.2018.09.007

[40] Hatakeyama D , Masuda T , Miki R , Ohtsuki S , Kuzuhara T . In‐vitro acetylation of SARS‐CoV and SARS‐CoV‐2 nucleocapsid proteins by human PCAF and GCN5. Biochem Biophys Res Commun. 2021;557:273‐279. 33894414 10.1016/j.bbrc.2021.03.173 PMC8030717

[41] Nakamura H , Kobayashi S , Kojima S , Urano A , Ito E . PKA‐dependent regulation of synaptic enhancement between a buccal motor neuron and its regulatory interneuron in *Lymnaea stagnalis* . Zoolog Sci. 1999;16:387‐394.

[42] Dhalluin C , Carlson JE , Zeng L , He C , Aggarwal AK , Zhou MM . Structure and ligand of a histone acetyltransferase bromodomain. Nature. 1999;399:491‐496. 10365964 10.1038/20974

[43] Mujtaba S , He Y , Zeng L , et al. Structural mechanism of the bromodomain of the coactivator CBP in p53 transcriptional activation. Mol Cell. 2004;13:251‐263. 14759370 10.1016/s1097-2765(03)00528-8

[44] Plotnikov AN , Yang S , Zhou TJ , Rusinova E , Frasca A , Zhou MM . Structural insights into acetylated‐histone H4 recognition by the bromodomain‐PHD finger module of human transcriptional coactivator CBP. Structure. 2014;22:353‐360. 24361270 10.1016/j.str.2013.10.021 PMC3923519

[45] Xu L , Cheng A , Huang M , et al. Structural insight into the recognition of acetylated histone H3K56ac mediated by the bromodomain of CREB‐binding protein. FEBS J. 2017;284:3422‐3436. 28815970 10.1111/febs.14198

[46] Delvecchio M , Gaucher J , Aguilar‐Gurrieri C , Ortega E , Panne D . Structure of the p300 catalytic core and implications for chromatin targeting and HAT regulation. Nat Struct Mol Biol. 2013;20:1040‐1046. 23934153 10.1038/nsmb.2642

[47] Ortega E , Rengachari S , Ibrahim Z , et al. Transcription factor dimerization activates the p300 acetyltransferase. Nature. 2018;562:538‐544. 30323286 10.1038/s41586-018-0621-1 PMC6914384

[48] Zhang Y , Xue Y , Shi J , et al. The ZZ domain of p300 mediates specificity of the adjacent HAT domain for histone H3. Nat Struct Mol Biol. 2018;25:841‐849. 30150647 10.1038/s41594-018-0114-9 PMC6482957

[49] Liu X , Wang L , Zhao K , et al. The structural basis of protein acetylation by the p300/CBP transcriptional coactivator. Nature. 2008;451:846‐850. 18273021 10.1038/nature06546

[50] Maksimoska J , Segura‐Peña D , Cole PA , Marmorstein R . Structure of the p300 histone acetyltransferase bound to acetyl‐coenzyme a and its analogues. Biochemistry. 2014;53:3415‐3422. 24819397 10.1021/bi500380f PMC4045318

[51] Sadamoto H , Takahashi H , Okada T , Kenmoku H , Toyota M , Asakawa Y . *De novo* sequencing and transcriptome analysis of the central nervous system of mollusc *Lymnaea stagnalis* by deep RNA sequencing. PLoS One. 2012;7:e42546. 22870333 10.1371/journal.pone.0042546 PMC3411651

[52] Mayr B , Montminy M . Transcriptional regulation by the phosphorylation‐dependent factor CREB. Nat Rev Mol Cell Biol. 2001;2:599‐609. 11483993 10.1038/35085068

[53] Radhakrishnan I , Perez‐Alvarado GC , Parker D , Dyson HJ , Montminy MR , Wright PE . Solution structure of the KIX domain of CBP bound to the transactivation domain of CREB: a model for activator:coactivator interactions. Cell. 1997;91:741‐752. 9413984 10.1016/s0092-8674(00)80463-8

[54] Shaywitz AJ , Dove SL , Kornhauser JM , Hochschild A , Greenberg ME . Magnitude of the CREB‐dependent transcriptional response is determined by the strength of the interaction between the kinase‐inducible domain of CREB and the KIX domain of CREB‐binding protein. Mol Cell Biol. 2000;20:9409‐9422. 11094091 10.1128/mcb.20.24.9409-9422.2000 PMC102197

[55] Liang G , Hai T . Characterization of human activating transcription factor 4, a transcriptional activator that interacts with multiple domains of cAMP‐responsive element‐binding protein (CREB)‐binding protein. J Biol Chem. 1997;272:24088‐24095. 9295363 10.1074/jbc.272.38.24088

[56] Gachon F , Devaux C , Mesnard JM . Activation of HTLV‐I transcription in the presence of tax is independent of the acetylation of CREB‐2 (ATF‐4). Virology. 2002;299:271‐278. 12202230 10.1006/viro.2002.1501

[57] Lassot I , Estrabaud E , Emiliani S , Benkirane M , Benarous R , Margottin‐Goguet F . p300 modulates ATF4 stability and transcriptional activity independently of its acetyltransferase domain. J Biol Chem. 2005;280:41537‐41545. 16219772 10.1074/jbc.M505294200

[58] Murata T , Kurokawa R , Krones A , et al. Defect of histone acetyltransferase activity of the nuclear transcriptional coactivator CBP in Rubinstein‐Taybi syndrome. Hum Mol Genet. 2001;10:1071‐1076. 11331617 10.1093/hmg/10.10.1071

[59] Chrivia JC , Kwok RP , Lamb N , Hagiwara M , Montminy MR , Goodman RH . Phosphorylated CREB binds specifically to the nuclear protein CBP. Nature. 1993;365:855‐859. 8413673 10.1038/365855a0

[60] Chatterjee S , Angelakos CC , Bahl E , et al. The CBP KIX domain regulates long‐term memory and circadian activity. BMC Biol. 2020;18:155. 33121486 10.1186/s12915-020-00886-1 PMC7597000

[61] Glass CK , Rose DW , Rosenfeld MG . Nuclear receptor coactivators. Curr Opin Cell Biol. 1997;9:222‐232. 9069256 10.1016/s0955-0674(97)80066-x

[62] Raisner R , Kharbanda S , Jin L , et al. Enhancer activity requires CBP/P300 bromodomain‐dependent histone H3K27 acetylation. Cell Rep. 2018;24:1722‐1729. 30110629 10.1016/j.celrep.2018.07.041

[63] Zeng L , Zhang Q , Gerona‐Navarro G , Moshkina N , Zhou MM . Structural basis of site‐specific histone recognition by the bromodomains of human coactivators PCAF and CBP/p300. Structure. 2008;16:643‐652. 18400184 10.1016/j.str.2008.01.010 PMC3339198

[64] Kojima S , Hosono T , Fujito Y , Ito E . Optical detection of neuromodulatory effects of conditioned taste aversion in the pond snail *Lymnaea stagnalis* . J Neurobiol. 2001;49:118‐128. 11598919 10.1002/neu.1069

[65] Yamanaka M , Hatakeyama D , Sadamoto H , Kimura T , Ito E . Development of key neurons for learning stimulates learning ability in *Lymnaea stagnalis* . Neurosci Lett. 2000;278:113‐116. 10643814 10.1016/s0304-3940(99)00916-7

[66] Sunada H , Lukowiak K , Ito E . Cerebral giant cells are necessary for the formation and recall of memory of conditioned taste aversion in *Lymnaea* . Zoolog Sci. 2017;34:72‐80. 28148214 10.2108/zs160152

[67] Nikitin ES , Vavoulis DV , Kemenes I , et al. Persistent sodium current is a nonsynaptic substrate for long‐term associative memory. Curr Biol. 2008;18:1221‐1226. 18701288 10.1016/j.cub.2008.07.030

[68] Vavoulis DV , Nikitin ES , Kemenes I , et al. Balanced plasticity and stability of the electrical properties of a molluscan modulatory interneuron after classical conditioning: a computational study. Front Behav Neurosci. 2010;4:19. 20485464 10.3389/fnbeh.2010.00019 PMC2871690

[69] Marra V , O'Shea M , Benjamin PR , Kemenes I . Susceptibility of memory consolidation during lapses in recall. Nat Commun. 2013;4:1578. 23481386 10.1038/ncomms2591 PMC3615469

[70] Scheibenstock A , Krygier D , Haque Z , Syed N , Lukowiak K . The soma of RPeD1 must be present for long‐term memory formation of associative learning in *Lymnaea* . J Neurophysiol. 2002;88:1584‐1591. 12364489 10.1152/jn.2002.88.4.1584

[71] Hatakeyama D , Fujito Y , Sakakibara M , Ito E . Expression and distribution of transcription factor CCAAT/enhancer‐binding protein in the central nervous system of *Lymnaea stagnalis* . Cell Tissue Res. 2004;318:631‐641. 15578275 10.1007/s00441-004-0965-8

[72] Ceseña TI , Cardinaux JR , Kwok R , Schwartz J . CCAAT/enhancer‐binding protein (C/EBP) β is acetylated at multiple lysines: acetylation of C/EBPβ at lysine 39 modulates its ability to activate transcription. J Biol Chem. 2007;282:956‐967. 17110376 10.1074/jbc.M511451200

[73] Hatakeyama D , Tierling S , Kuzuhara T , Müller U . Epigenetic regulation of gene expression in the nervous system. In: Oka K , Ogawa H , eds. Methods in Neuroethological Research. Springer; 2013:151–171.

[74] Coste C , Gérard N , Dinh CP , et al. Targeting MHC regulation using polycyclic polyprenylated acylphloroglucinols isolated from *Garcinia bancana* . Biomolecules. 2020;10:1266. 32887413 10.3390/biom10091266 PMC7563419

[75] Monsey MS , Sanchez H , Taylor JR . The naturally occurring compound *Garcinia indica* selectively impairs the reconsolidation of a cocaine‐associated memory. Neuropsychopharmacology. 2017;42:587‐597. 27380937 10.1038/npp.2016.117 PMC5240167

[76] Maddox SA , Watts CS , Schafe GE . p300/CBP histone acetyltransferase activity is required for newly acquired and reactivated fear memories in the lateral amygdala. Learn Mem. 2013;20:109‐119. 23328899 10.1101/lm.029157.112 PMC3549061

[77] Ahmed T , Gilani AH , Hosseinmardi N , Semnanian S , Enam SA , Fathollahi Y . Curcuminoids rescue long‐term potentiation impaired by amyloid peptide in rat hippocampal slices. Synapse. 2011;65:572‐582. 20963814 10.1002/syn.20876

[78] Zhang L , Fang Y , Xu Y , et al. Curcumin improves amyloid β‐peptide (1‐42) induced spatial memory deficits through BDNF‐ERK signaling pathway. PLoS One. 2015;10:e0131525. 26114940 10.1371/journal.pone.0131525 PMC4482657

[79] Liu D , Wang Z , Gao Z , et al. Effects of curcumin on learning and memory deficits, BDNF, and ERK protein expression in rats exposed to chronic unpredictable stress. Behav Brain Res. 2014;271:116‐121. 24914461 10.1016/j.bbr.2014.05.068

[80] Namgyal D , Ali S , Mehta R , Sarwat M . The neuroprotective effect of curcumin against cd‐induced neurotoxicity and hippocampal neurogenesis promotion through CREB‐BDNF signaling pathway. Toxicology. 2020;442:152542. 32735850 10.1016/j.tox.2020.152542

[81] Belviranli M , Okudan N , Esra K , Atalik N , Öz M . Curcumin improves spatial memory and decreases oxidative damage in aged female rats. Biogerontology. 2013;14:187‐196. 23609199 10.1007/s10522-013-9422-y

[82] Cheng YF , Guo L , Xie YS , et al. Curcumin rescues aging‐related loss of hippocampal synapse input specificity of long term potentiation in mice. Neurochem Res. 2013;38:98‐107. 23011209 10.1007/s11064-012-0894-y

[83] Tang H , Lu D , Pan R , Qin X , Xiong H , Dong J . Curcumin improves spatial memory impairment induced by human immunodeficirncy virus type i glycoprotein V3 loop peptide in rats. Life Sci. 2009;85:1‐10. 19345695 10.1016/j.lfs.2009.03.013

[84] Shen LL , Jiang ML , Liu SS , et al. Curcumin improves synaptic plasticity impairment induced by HIV‐1gp120 V3 loop. Neural Regen Res. 2015;10:925‐931. 26199609 10.4103/1673-5374.158358 PMC4498354

