# Supplementary material for: Molecular and functional characterization of an evolutionarily conserved CREB‐binding protein in the Lymnaea CNS
Source: FASEB J. 2022 Oct 17;36(11):e22593. doi: 10.1096/fj.202101225RR (PMC9828244; doi:10.1096/fj.202101225RR)
Supplement: Supplementary file 1 — Figure S1 [file FSB2-36-0-s001.pdf]

Supplemental information

Molecular and functional characterization of an evolutionarily conserved CREB-binding protein in the *Lymnaea* CNS

Dai Hatakeyama[1,2] | Hiroshi Sunada[3] | Yuki Totani[4] | Takayuki Watanabe[5] | Ildikó Felletár[1] | Adam Fitchett[1] | Murat Eravci[1] | Aikaterini Anagnostopoulou[1] | Ryosuke Miki[2] | Ayano Okada[2] | Naoya Abe[2] | Takashi Kuzuhara[2] | Ildikó Kemenes[1] | Etsuro Ito[3,4] | György Kemenes[1]

[1] Sussex Neuroscience, School of Life Sciences, University of Sussex, Brighton BN1 9QG, UK.

[2] Faculty of Pharmaceutical Sciences, Tokushima Bunri University, Tokushima 770-8514, Japan.

[3] Kagawa School of Pharmaceutical Sciences, Tokushima Bunri University, Sanuki 769-2193, Japan.

[4] Department of Biology, Waseda University, Tokyo 162-8480, Japan.

[5] Laboratory of Neuroethology, Sokendai-Hayama, Hayama 240-0193, Japan.

MATERIALS AND METHODS

Molecular cloning of LymCBP

To clone LymCBP, a series of degenerate PCR was performed with TaKaRa Ex Taq® (Takara Clontech) and primers, which were designed at the basis of highly conserved domains, such as Taz1 (transcriptional adapter zinc-binding 1), KIX domain and Taz2 domain, of *Aplysia* CBP (ApCBP; GenBank accession number: AY064470). After the sequential analyses of the Taz1, KIX and Taz2 domains of LymCBP, we performed 5' and 3'RACE, and the PCR for the internal region between Taz1 and KIX domains and between KIX and Taz2 domains. The FirstChoice® RLM-RACE Kit (ThermoFisher) was used for amplification of 5' and 3' ends of LymCBP. All PCR products were subcloned with TA Cloning® Kit (ThermoFisher) or pGEM®-T Easy Vector System (Promega). Nucleotide sequences of primers were summarized in Table S1 (No. 1-14).

Phylogenetic tree of LymCBP

To construct a phylogenetic tree of the CBP/p300 proteins, we aligned the full-length deduced amino acid sequence of the *Lymnaea* CBP with those of the known homologs of other species (listed with each Accession Number in Table 2) by using the MUSCLE algorisms on the Geneious (v9.1) program (available from http://www.geneious.com/).[1] Maximum likelihood tree was constructed from the aligned sequences using the MEGA 6 program with default settings of the program.[2] Thousand bootstrap replications were conducted to evaluate the reliabilities of the reconstructed trees. The obtained tree was visualized with the FigTree (v1.4.2) program (available from http://tree.bio.ed.ac.uk/software/figtree/). A CBP homolog of the Choanoflagellate *Salpingoeca rosetta* was used as an outgroup.

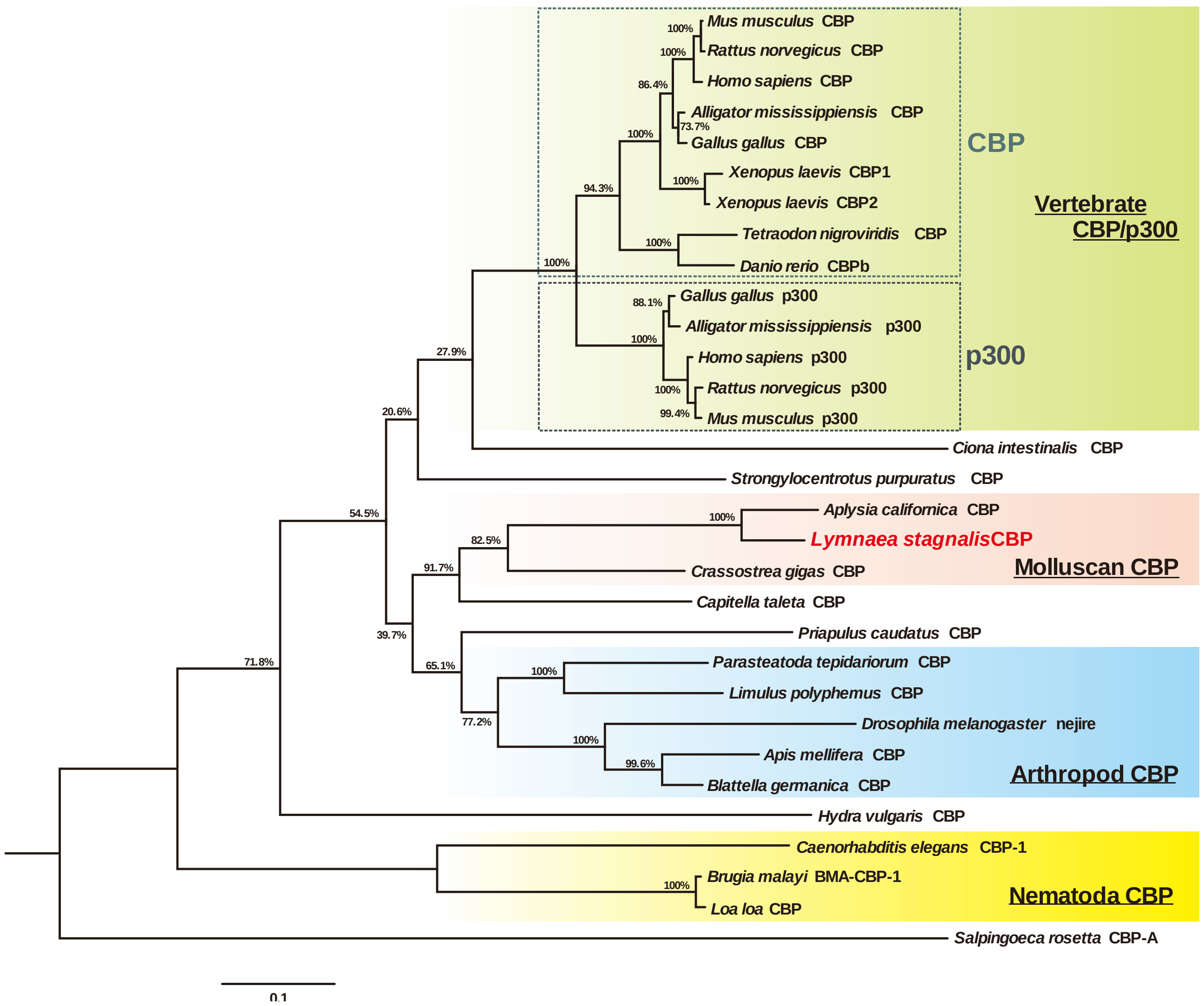

FIGURE S1. Molecular phylogenetic tree of *Lymnaea* CBP and other p300/CBP proteins. The scale bar indicates 0.1 substitutions per site. Bootstrap values are shown at the nodes. A CBP homologue of the Choanoflagellate *Salpingoeca rosetta* was used as an outgroup. The GenBank accession numbers of p300/CBP-like proteins are listed in **Table S1**.

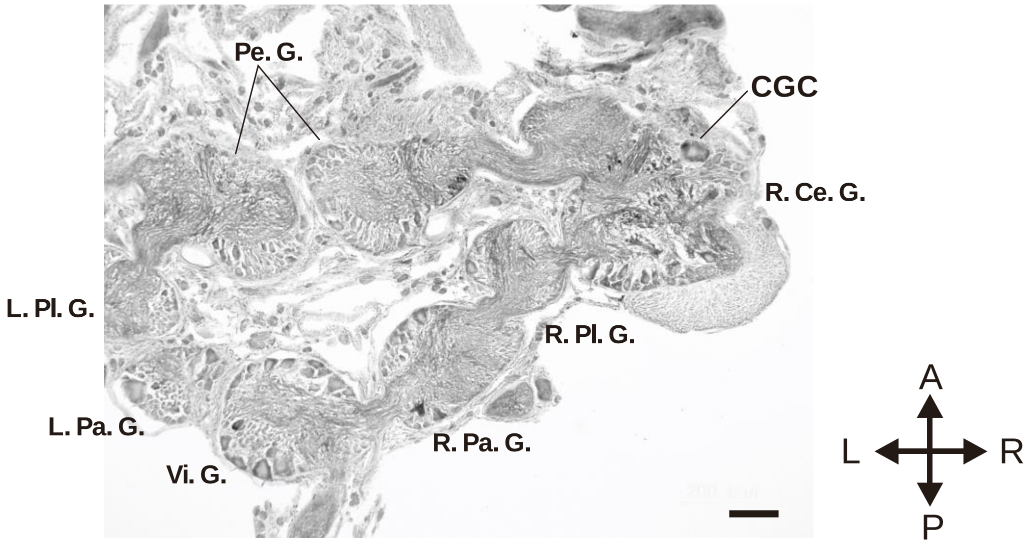

FIGURE S2. Negative control of *in-situ* hybridization using a sense probe for LymCBP. The background staining was equally observed around the tissue of *Lymnaea* CNS. Scale bar = 200 μm. CGC: Cerebral Giant Cell, Pe. G.: pedal ganglion, Pl. G.: pleural ganglion, Pa. G.: parietal ganglion, Vi. G.: visceral ganglion, Ce. G.: cerebral ganglion, L: left, R: right, A: anterior, and P: posterior.

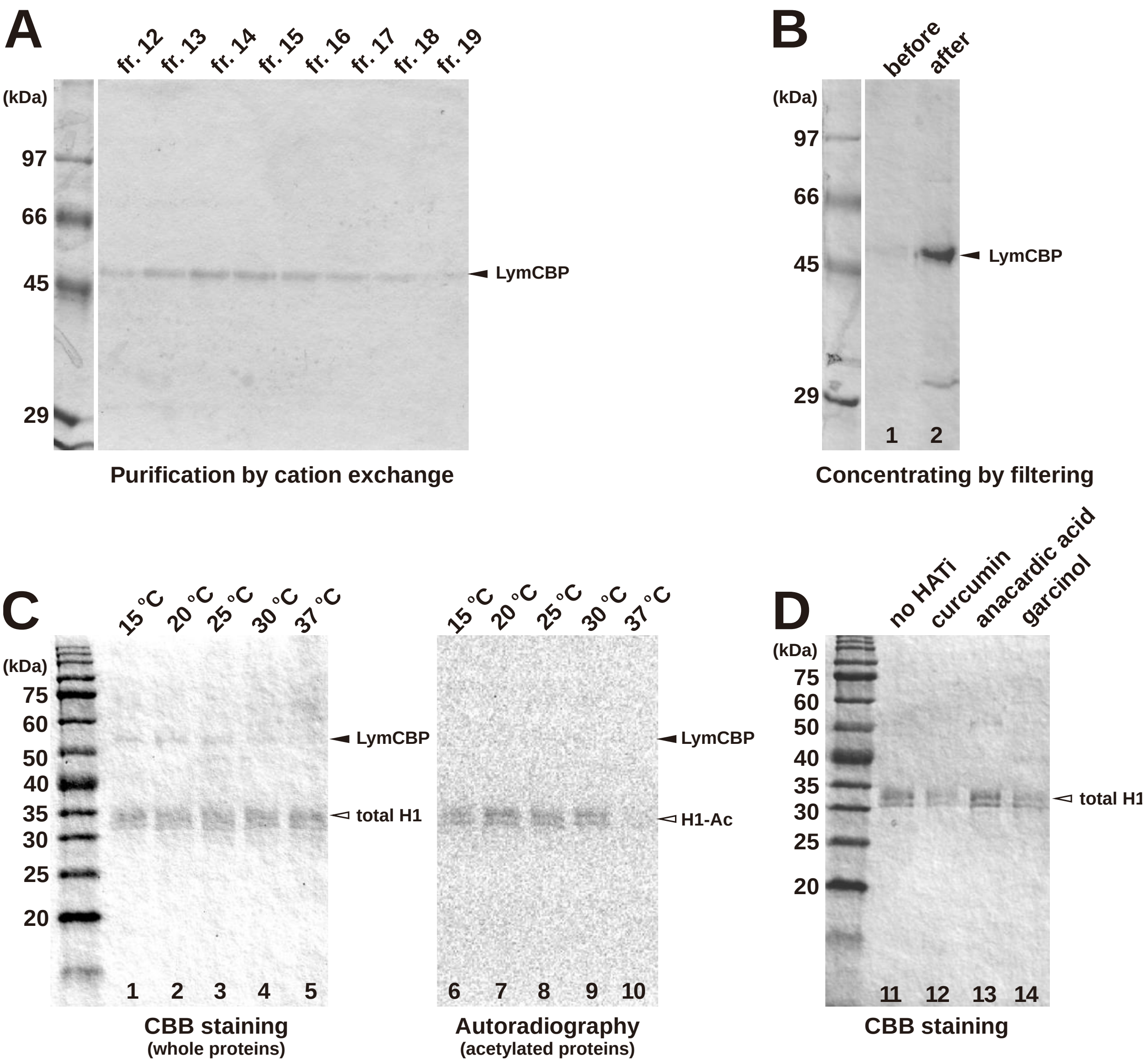

FIGURE S3. Production of a partial recombinant protein of the LymCBP HAT domain for *in vitro* histone H1 acetylation assays in the absence and presence of HAT inhibitors. (A) After purification by cation exchange, the partial recombinant protein was detected in fractions No. 12–19 (bands shown with arrowhead). The concentrations of these preparations were too low to be measured by Bradford protein assay. (B) All fractions obtained by cation-exchange chromatography were pooled (lane 1), and the recombinant protein was concentrated using a centrifugal filter. A high enough concentration of the recombinant protein for biochemical experiments was obtained (lane 2). (C) Determination of the incubation temperature for the *in vitro* acetylation assay. Left panel: Coomassie Brilliant Blue (CBB) staining detected both the recombinant LymCBP HAT domain and total histone H1 in the temperature range 15ºC to 30ºC but the recombinant LymCBP HAT domain was not detected at 37ºC. Right panel: The acetylation assay shows a similar temperature dependence of the detection of acetylated H1 to that of the recombinant LymCBP HAT domain in the left panel. (D) Total histone H1 can be detected after incubation with HAT inhibitors. Although the signal is weaker in the curcumin and garcinol lanes compared to the other two lanes, there is no substantial degradation of total H1 by the HAT inhibitors.

A

	mammal	*Lymnaea*	Ref.
mutant 1	R1379P	R1300P	57
mutant 2	I1456A	I1376A	58
mutant 3	D1522A	D1442A	58
mutant 4	Y1540A/ F1541A	Y1460A/ F1461A	9

B

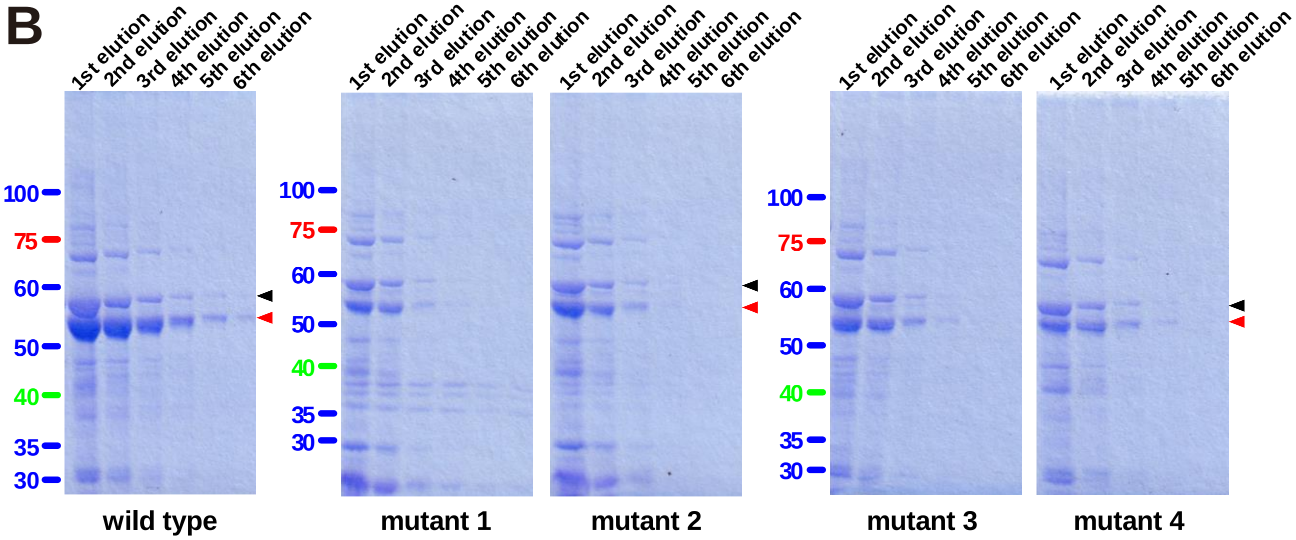

C

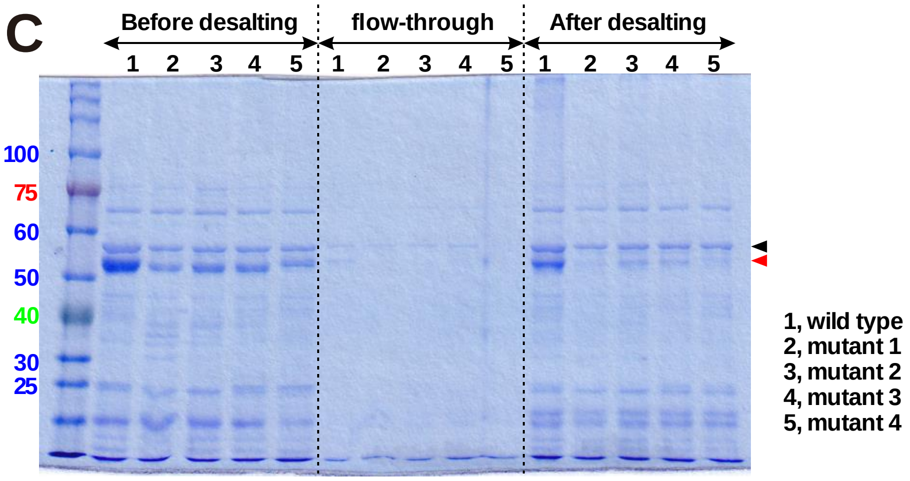

FIGURE S4. Producing mutant recombinant proteins of the LymCBP HAT domain. (A) List of lysine residues which were suggested to be effective for HAT activity of LymCBP. (B) The partial recombinant protein of LymCBP HAT domain purified using the Ni-NTA agarose resin was detected (red arrowheads). Expression levels of the 4 mutant recombinant proteins were lower than that of the wild-type recombinant protein. The black arrowheads indicate the contaminated protein derived from *E. coli* cells. (C) These recombinant proteins were treated with the PD-10 desalting column. After this treatment, bands of the 4 mutant recombinant proteins were much weaker and their concentration of was insufficient for purification by cation-exchange chromatography..

A

	mammal	*Lymnaea*	Ref.
mutant 5	Y1467A	Y1387A	58
mutant 6	H1471A	H1391A	58

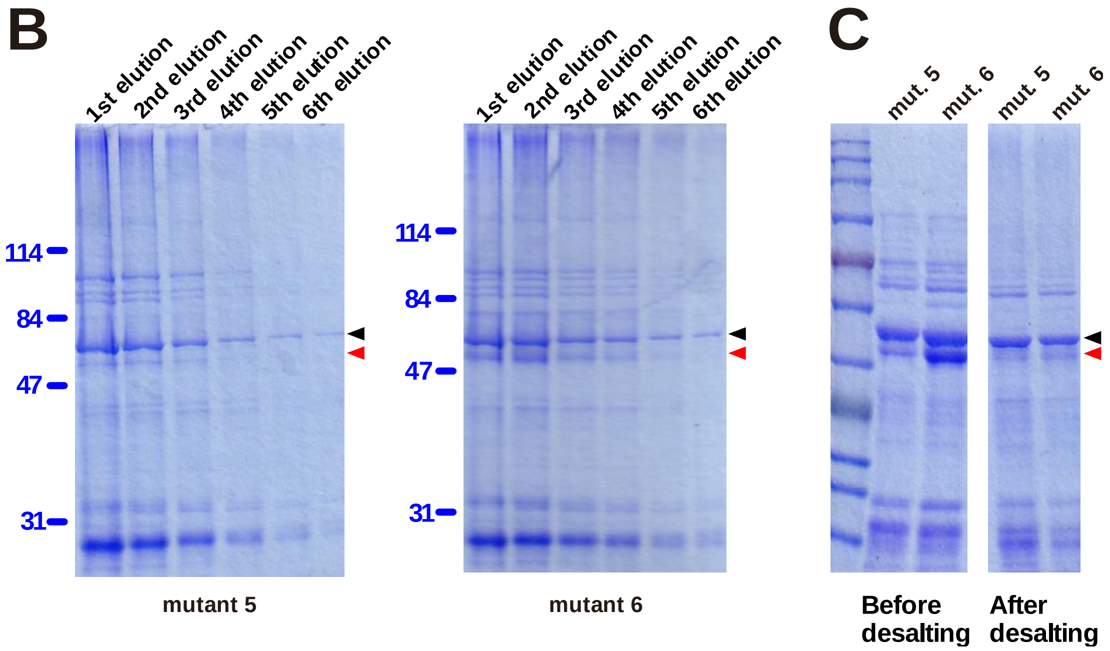

FIGURE S5. Producing recombinant proteins of LymCBP HAT domain containing other amino-acid substitutions. (A) List of lysine residues which were suggested to be effective for HAT activity of LymCBP. (B) The partial recombinant proteins of LymCBP HAT domain purified using the Ni-NTA agarose resin were detected (red arrowheads). Expression levels of the 2 mutant recombinant proteins were lower than that of the wild-type recombinant protein. The black arrowheads showed the contaminated protein derived from *E. coli* cells. (C) These recombinant proteins were treated with the PD-10 desalting column. After this treatment, bands of the 2 mutant recombinant proteins were much weaker, and their concentration was insufficient for purification by cation-exchange chromatography.

TABLE S1. Nucleotide sequences of primers and probes used for cloning. (No. 1-14), qRT-PCR (No. 15-20), synthesis for *in situ* hybridization probe (No. 21, 22), synthesis for LymCBP recombinant proteins (No. 23-32). Amino acid sequences of ApCBP for designing degenerate PCR (DGPCR) was shown with nucleotide sequences.

	Names of primers and probes	Nucleotide sequences
1.	DGPCR_Taz1_fw	5'-CTS ATC CAG CAG CAG CTB GTB CTS C-3' (LIQQQLVL)
2.	DGPCR_Taz1_rv	5'-ATT TGW CGA GAW GAR GCA CAR TGR G-3' (AHCASSRQII)
3.	DGPCR_KIX_fw	5'-CTG GTS CAR GCY ATY TTY CC-3' (KLVQAIFP)
4.	DGPCR_KIX_rv	5'-GTA SAT YTT CTC RGC SAG CAA RTG-3' (HLLAEKIY)
5.	DGPCR_Taz2_fw	5'-TCC ATT CAR CGC TGY ATC TCN TCH C-3' (SIQRCISSL)
6.	DGPCR_Taz2_rv	5'-ACA GAA TGG GAC CKG RCA YTT RTT YTC-3' (ENKCQVPFC)
7.	5'RACE_outer	5'-TTA CTG GCT GGT ATA GAT TTA ACA ATA TCT GGA GC-3'
8.	5'RACE_inner	5'-TAT GTT ACC CAA AAG ATT ACT CAT ATG ACT GCT AGG-3'
9.	3'RACE_outer	5'-CTA ACT GTC GTA TCA ACT CGT GCA TCA AG-3'
10.	3'RACE_inner	5'-GCA TCA AGA TGA AAC GTG TTG TTT CAC-3'
11.	Taz1-KIX_fw	5'-ACA TGC AAC AAA GGA AAA TCT TG-3'
12.	Taz1-KIX_rv	5'-TGA TAA TAT TCT CCT CTA TTG TTA GCA G-3'
13.	KIX-Taz2_fw	5'-ACA TGC AAC AAA GGA AAA TCT TGT GAA GTG GCC C-3'
14.	KIX-Taz2_rv	5'-CGT ACA ATG TTT GGC ATG GTA ACA ACA GAG TGC-3'
15.	qPCR_cloning_fw	5'-GAT GTA AAC ATG GCC GAC CAC CAA GTT G-3'
16.	qPCR_cloning_rv	5'-AGA CGA TGT AGC AAC TTT GTT TGT ATT GGC-3'
17.	qPCR_RT	5'-TGT TAA GAG ACA TCG GCA TTG ATG-3'
18.	qPCR_fw	5'-GCC CTC CGG CCA ACA AGA A-3'
19.	qPCR_rv	5'-TAT TAT CGC TGG GTG TAT TGA GAG AT-3'
20.	qPCR_probe	5'-[Fam]-ACC CAG AAT CGG ATC AC-[Tamra]-3'
21.	in-situ-probe_fw	5'-**GAT ATC** TCT TGA AAT CTT AGT GCT GTG TTG ATG-3' (***Eco*R V**)
22.	in-situ-probe_rv	5'-**GGA TCC** ATC AGC AGA AGT CTG TCG AGT ACC-3' (***Bam*H I**)
23.	recombinant_fw	5'-CGC GGC AGC CAT ATG **GCT AGC** AGA CGA AAA GAA AAC AAA TTT ACA GCC-3' [nucleotide sequence of pET28a(+) plasmid, **the restriction site of *Nhe* I**, nucleotide sequence of LymCBP]
24.	recombinant_rv	5'-ACG GAG CTC GAA TTC **GGA TCC** TTA ATC AGG TTC TGT GAT TGG TGG CAG-3' [nucleotide sequence of pET28a(+) plasmid, **the restriction site of *Bam*H I**, nucleotide sequence of LymCBP]
25.	R1300P-ole-fw	5'-CCA TTA ATG AAA AAG CCA TTT GGT GAT GAG ATA CC-3'
26.	R1300P-ole-rv	5'-GGT ATC TCA TCA CCA AAT GGC TTT TTC ATT AAT GG-3'
27.	I1376A-ole-fw	5'-TAT CAT GAA CTC CTC GCT GGC TAT CTT GAA TAT G-3'
28.	I1376A-ole-rv	5'-CAT ATT CAA GAT AGC CAG CGA GGA GTT CAT GAT A-3'
29.	D1442A-ole-fw	5'-GTA GTA GAC TAC AAA GCC ATT TTT AAA GAT GC-3'
30.	D1442A-ole-rv	5'-GCA TCT TTA AAA ATG GCT TTG TAG TCT ACT AC-3'
31.	Y1460A-F1461A-ole-fw	5'-CAA AAG ACA TGG CAG CCG CTG AGG GAG ACT TTT G-3'
32.	Y1460A-F1461A-ole-rv	5'-CAA AAG TCT CCC TCA GCG GCT GCC ATG TCT TTT G-3'

TABLE S2. GenBank accession numbers of the various CBP and p300 structures used for the molecular phylogenetic tree shown in Fig. S2.

Species	Protein	GenBank Accession No.
Alligator mississippiensis	CBP	XP_006264860
Alligator mississippiensis	p300	XP_014463094
Apis mellifera	CBP	XP_006568897
Aplysia californica	CBP	NP_001191640.1
Blattella germanica	CBP	CUT08824
Brugia malayi	CBP	CTP80907
Caenorhabditis elegans	CBP	NP_499161
Capitella teleta	CBP	ELU13629
Ciona intestinalis	CBP	XP_009860762.1
Crassostrea gigas	CBP	XP_011427368.1
Danio rerio	CBP	XP_009297440.1
Drosophila melanogaster	nejire	NP_001188575.1
Gallus gallus	CBP	XP_015150108.1
Gallus gallus	p300	XP_004937767
Homo sapiens	CBP	NP_004371.2
Homo sapiens	p300	NP_001420
Hydra vulgaris	CBP	XP_002156492.2
Hymenolepis microstoma	CBP	CDS30104
Limulus polyphemus	CBP	XP_013776092
Loa loa	CBP	EJD74639
Lymnaea stagnalis	CBP	AB217914
Mus musculus	CBP	NP_001020603.1
Mus musculus	p300	NP_808489
Opisthorchis viverrini	CBP	XP_009171514
Parasteatoda tepidariorum	CBP	XP_015922458
Priapulus caudatus	CBP	XP_014664166
Rattus norvegicus	CBP	NP_596872.3
Rattus norvegicus	p300	XP_017450856
Salpingoeca rosetta	CBP	XP_004998720
Schistosoma mansoni	CBP	CCD78413
Strongylocentrotus purpuratus	CBP	XP_011677827.1
Takifugu rubripes	CBP	XP_011602271.1
Xenopus laevis	CBP1	NP_001088637.1
Xenopus laevis	CBP2	NP_001192159.1

TABLE S3. List of functional amino acid residues conserved in the bromodomain between human CBP and LymCBP. Different amino acid residues between proteins are highlighted with a shaded box.

human CBP	LymCBP
P 1110	P 999
F 1111	F 1000
V 1115	V 1004
L 1120	L 1009
G 1121	H 1010
I 1122	I 1011
P 1123	P 1012
D 1124	D 1013
Y 1125	Y 1014
I 1128	I 1017
L 1166	L 1055
Y 1167	Y 1056
N 1168	N 1057
R 1169	R 1058
V 1174	V 1063

TABLE S4. List of functional amino acid residues conserved in the RING domain and the HAT domain among human p300, human CBP and LymCBP. Different amino acid residues are highlighted with shaded boxes.

	human p300	human CBP	LymCBP
RING domain	Q 1173	Q 1209	Q 1100
	T 1174	T 1210	V 1101
	L 1182	L 1218	L 1109
	T 1184	T 1220	T 1111
	E 1242	E 1278	D 1191
HAT domain	E 1640	E 1677	E 1607
	R 1645	R 1682	R 1612
	R 1646	R 1683	R 1613

TABLE S5. List of functional amino acid residues essential for interacting with acetyl-CoA conserved in the HAT domain among human p300, human CBP and LymCBP.

human p300	human CBP	LymCBP
I 1395	I 1431	I 1351
L 1398	L 1434	L 1354
R 1410	R 1446	R 1366
I 1435	I 1471	I 1391
W 1436	W 1472	W 1392
K 1456	K 1492	K 1412
R 1462	R 1498	R 1418
Y 1467	Y 1503	Y 1423

TABLE S6. List of functional amino acid residues essential for interacting with histones conserved in the ZZ domain among human p300, human CBP and LymCBP. Different amino acid residues are highlighted with shaded boxes.

human p300	human CBP	LymCBP
D 1664	D 1701	D 1631
R 1665	R 1702	A 1632
F 1666	F 1703	F 1633
T 1669	T 1706	T 1636
N 1671	N 1708	N 1638
E 1687	E 1724	D 1654
D 1688	D 1725	D 1655
D 1690	D 1727	D 1627

TABLE S7. List of functional amino acid residues in the HAT domains essential for interacting with garcinol among human p300, human CBP and LymCBP.

human p300	human CBP	LymCBP
S 1400	S 1436	S 1356
Y 1414	Y 1450	Y 1370
D 1444	D 1480	D 1400
Y 1446	Y 1482	Y 1402
Q 1455	Q 1491	Q 1411

REFERENCES

1. Edgar RC. MUSCLE: multiple sequence alignment with high accuracy and high throughput. *Nucleic Acids Res* 2004;32:1792–1797.
2. Tamura K, Stecher G, Peterson D, Filipski A, Kumar S. MEGA6: Molecular Evolutionary Genetics Analysis version 6.0. *Mol Biol Evol* 2013;30:2725–2729.
